# Limited interactions between space- and feature-based attention in visually sparse displays

**DOI:** 10.1167/jov.20.4.5

**Published:** 2020-04-09

**Authors:** Guangsheng Liang, Miranda Scolari

**Affiliations:** Department of Psychological Sciences, Texas Tech University, Lubbock, TX, USA

**Keywords:** space-based attention, feature-based attention, perceptual decision making, EZ-diffusion model

## Abstract

Top-down visual attention selectively filters sensory input so relevant information receives preferential processing. Feature-based attention (FBA) enhances the representation of relevant low-level features, whereas space-based attention (SBA) enhances information at relevant location(s). The present study investigates whether the unique influences of SBA and FBA combine to facilitate behavior in a perceptually demanding discrimination task. We first demonstrated that, independently, both color and location pre-cues could effectively direct attention to facilitate perceptual decision making of a target. We then examined the combined effects of SBA and FBA in the same design by deploying a predictive color arrow pre-cue. Only SBA effects were observed in performance accuracy and reaction time. However, we detected a reaction time cost when a valid spatial cue was paired with a feature cue. A computational perceptual decision-making model largely provided converging evidence that contributions from FBA were restricted to facilitating the speed with which the relevant item was identified. Our results suggest that both selection mechanisms can be used in isolation to resolve a perceptually challenging target in a sparse display, but with little additive perceptual benefit when cued simultaneously. We conclude that there is at least some higher order interdependence between space-based and feature-based selection during decision making under specific conditions.

## Introduction

Covert visual attention filters sensory input, where desired input receives preferential processing, and ultimately improves the perception of selected stimuli. This selection process can occur in several different ways. A space-based attention (SBA) mechanism enhances the representation of stimuli at selected spatial locations and suppresses responses outside the selected region (Carrasco, 2006; Moran & Desimone, 1985; Posner, 1980; Reynolds & Chelazzi, 2004). In contrast, feature-based attention (FBA) enhances the representation of selected feature values (e.g., within color, motion direction, or orientation space) throughout the visual field regardless of target location while suppressing unselected feature values (Liu & Mance, 2011; Motter, 1994; Rossi & Paradiso, 1995; Serences & Boynton, 2007; Treue & Trujillo, 1999; White & Carrasco, 2015). Both SBA and FBA have been studied extensively in isolation, leading to undisputed evidence that they are operationally distinct. For example, SBA effects are present even when only the expected location—but not the specific feature(s)—of an upcoming target are known (e.g., Awh, Matsukura, & Serences, 2003; Awh, Sgarlata, & Kliestik, 2005; Desimone & Duncan, 1995; Reynolds & Chelazzi, 2004), and FBA effects are clearly present in studies using spatially superimposed relevant and irrelevant features such that SBA is of no use at all (e.g., Corbetta, Miezin, Dobmeyer, Shulman, & Petersen, 1991; Lankheet & Verstraten, 1995; see also Scolari, Ester, & Serences, 2014).

Space- and feature-based selection have separately been shown to exert similar influences both on the perception of relevant stimuli and on relevant underlying neural populations. Perceptually, both selection mechanisms seem to elevate the subjective salience of target stimuli relative to distracting input (Carrasco, 2011; Lankheet & Verstraten, 1995; Liu, Abrams, & Carrasco, 2009; White & Carrasco, 2011). Liu et al. (2009), for example, demonstrated that stimuli presented at an endogenously attended location were perceived to have higher contrast than physically matched stimuli at an unattended location. Similarly, Lankheet and Verstraten (1995) found motion after-effects generated from superimposed random dot kinetograms were significantly stronger for an attended direction of motion compared with a spatially overlapping unattended direction of motion. Both SBA- and FBA-driven perceptual changes likely result from corresponding response modulations in specific sensory populations within visual cortex (Haenny & Schiller, 1988; Hayden & Gallant, 2009; Moran & Desimone, 1985; Treue & Martinez-Trujillo, 1999). Together, the behavioral and physiologic findings logically lead to investigations into possible interactions between both selection mechanisms. When studied in conjunction, however, broad investigations have led to divergent conclusions on whether and how the two selection mechanisms interact. This in turn leads to conflicting accounts as to whether space- and feature-based selection are deployed via common or independent mechanisms.

One prospective supported by neuroimaging studies proposes that SBA and FBA operate in parallel (Andersen, Fuchs, & Müller, 2011; Boynton, 2005; Cohen & Maunsell, 2011; Egner et al., 2008). Although common frontal, parietal, and cingulate regions have been shown to exhibit both SBA and FBA modulation, interactions between mechanisms were not observed, suggesting that distinct subregions within the structures may be responsible for domain-specific forms of attention (Egner et al., 2008; Greenberg, Esterman, Wilson, Serences, & Yantis, 2010; Wager, Jonides, & Reading, 2004). Physiology studies have similarly demonstrated that both SBA and FBA independently affect area V4 (Hayden & Gallant, 2009; McAdams & Maunsell, 2000). In sum, the evidence presented here suggests that the operation and magnitude of each attention effect is unchanged whether or not the other is simultaneously deployed.

A competing perspective proposes that SBA and FBA interact as a result of a common control mechanism (Ibos & Freedman, 2016; Patzwahl & Treue, 2009), although the exact nature of this interaction seems to be inconsistent at times. When both relevant space and feature information are cued, the extent to which relevant feature-matching distractors affect performance accuracy can depend on their spatial distance from targets (Leonard, Balestreri, & Luck, 2015). Other behavioral, neuroimaging, and electroencephalographic studies have produced converging evidence that FBA does not always operate globally, but may be restricted to attended regions of space (Bengson, Lopez-Calderon, & Mangun, 2012; Soto & Blanco, 2004; Stoppel et al., 2007). In contrast, when feature selection is necessary to constrain the search space, FBA precedes SBA (Kwak & Egeth, 1992; Shih & Sperling, 1996). This finding is particularly evident in cases of visual search, where attention is directed to the specific location(s) containing the relevant feature (Hamker, 2004; Hopf, Boelmans, Schoenfeld, Luck, & Heinze, 2004; Lim & Sinnett, 2014; Moore & Egeth, 1998). Taken together, these studies suggest that FBA depends on SBA when both feature and location are concurrently cued, and guides it when feature information can be used to localize a target. In either case, the effect of one may be dependent on the other.

White et al. (2015) recently attempted to reconcile the two divergent perspectives. They investigated how top-down SBA and FBA jointly influence perception in a superimposed dot pattern task. In their design, participants were provided with an integrated endogenous spatial and color cue before the onset of two overlapping dot patterns in each of four quadrants, defined by color (one of which was relevant). The spatial cue could be used to decrease the search space to a single visual field, whereas the feature cue could further decrease the search space to a particular set of colored dots within the attended field. Notably, although many studies providing support for dependent models have used RT evidence (Bengson et al., 2012; Kingstone, 1992), they largely focused on performance accuracy and limited the explanatory power of RT by design—arguing that RTs reflect a combination of attentional modulation and decision-related processes. When the salience of a subset of distracting dot patterns matched that of the target, attention effects in accuracy were super-additive; when only the target dot pattern was salient, attention effects were additive. Using a novel computational model, they demonstrated that, although behavioral effects point toward dependency between SBA and FBA only in the presence of highly salient distractors, the two selection mechanisms are best modeled as independent systems that jointly impact stimulus competition. Furthermore, they concluded that, when deployed together, SBA and FBA additively enhance relevant visual signals.

Although White et al. (2015) made important research gains, the conclusions may nonetheless be specific to certain task conditions and analysis approaches. The aggregate results across the studies presented here, after all, suggest that different task demands may induce different patterns of attentional modulation. Even among studies exploring interactions between SBA and FBA, the collection of methodologies and behavioral task designs are largely heterogeneous, which may impact how selective attention operates. Some studies that provided probable spatial and feature information of an upcoming target display did so via a single, integrated cue (e.g., Stoppel et al., 2007; White, Rolfs, & Carrasco, 2015); others provided two unique cues simultaneously (e.g., Bengson et al., 2012; Egner et al., 2008). Second, and perhaps more important, many of the studies cited included tasks in which an accurate behavioral response was directly predicted by the space and/or feature cue (e.g., Ibos & Freedman, 2016; Patzwahl & Treue, 2009), whereas another subset of studies included simultaneous distractors that could only be filtered out via use of specific cue(s) (e.g., Leonard et al., 2015; White et al., 2015). Delineating the conditions under which interactions are expected—and the nature of the interaction—is critical to fully understanding the mechanics of selective attention. Given that top-down attentional deployment seems to be at least partially flexible (Barbot & Carrasco, 2017; Lambert & Hockey, 1986; Scolari et al., 2014); however, demonstrations of combined and/or independent effects when either both forms of selection are necessary or each uniquely facilitates performance may not warrant broad generalizations beyond similar task environments.

In the current study, we set out to design a task in which both a space-based or feature-based selection strategy would be equally and redundantly viable to facilitate target identification in a sparse display, defined here as a single target and widely separated, distinctly colored distractors. Briefly, a colored relevant square was presented alone in one of four quadrants and the remaining three quadrants each contained a lone, uniquely colored distractor square. Crucially, the to-be-reported target—a gap on one of two vertical edges of the relevant square—was designed to be entirely orthogonal to the space and feature information provided by the pre-cue. Experiment 1 was first implemented to verify that attention effect magnitudes were equivalent when only a single spatial cue or single feature cue were provided during the task. Next, Experiment 2 used a central color arrow pre-cue that simultaneously provided the probable location and color of the relevant item. Here, each component of the cue predicted the related dimension of the relevant item (i.e., location or color) with equivalent frequency; in a small proportion of trials, one component accurately cued the relevant item and, equally often, both components were inaccurate. Therefore, deployment of either one selection mechanism at the exclusion of the other should equivalently facilitate performance. Simultaneous, independent deployments should afford a greater behavioral benefit simply by virtue of increasing the likelihood that the relevant square is attended, and even more so if such deployments are super-additive.

Importantly, in cases where the pre-cue was valid on both dimensions, space- and feature-based selection would redundantly decrease the search space to the single relevant item. Thus, interaction effects in performance accuracy could indicate dependency between mechanisms specifically within perceptual resolution of the target gap. Similar effects in RT may indicate 1) a speeded benefit for selecting the relevant square among three distinct distractors without a concurrent effect within target signal enhancement and/or 2) a speeded benefit while accumulating evidence in favor of one of the two response alternatives, likely involving signal enhancement. In an attempt to dissociate these possibilities, we implemented a robust EZ-diffusion model (a simple modification of the Ratcliff model; Ratcliff, 1978; Ratcliff & McKoon, 2007; van Ravenzwaaij, Donkin, & Vandekerckhove, 2017; Wagenmakers, Van Der Maas, & Grasman, 2007; Wagenmakers, Van Der Mass, Dolan, & Grasman, 2008). This cognitive model describes behavior in a two-alternative forced choice task via three unobserved estimates: 1) the rate of information accumulation in favor of one response alternative over another (drift rate), 2) the relative amount of information required to elicit a perceptual decision response (boundary separation). and 3) the remaining portion of RT that does not include evidence accumulation (non-decision time).

In summary, the design of this study allows us to address two important open questions regarding dual deployment of SBA and FBA. First, we investigate whether the two selection mechanisms combine in an independent or dependent manner to resolve a single target when the perceptual effects of attention are largely restricted to signal enhancement. Note that dependency can be either super-additive, where the effect of deploying one selection mechanism is magnified by deployment of the other; or sub-additive, where deploying one selection mechanism precludes deployment of the other. Second, using the diffusion model, we can take a more nuanced view of our data, allowing us to look for cueing effects and their possible interactions at several stages of the perceptual decision-making process.

## Experiment 1

### Methods

#### Participants

Thirty undergraduate students (8 males), naïve to the purposes of the study, were recruited via the Texas Tech University Research Participation System (SONA). Given a between-participants design, we elected to use a sample size on the high end of a broad range (3–30 participants; *M* = 14.19) reported in similar behavioral studies cited here (e.g., Leonard et al., 2015; Lim & Sinnett, 2014; Liu, Stevens, & Carrasco, 2007; White et al., 2015). Each participant gave written informed consent in accordance with both the requirements of the institutional review board and the Declaration of Helsinki, and received course credit for their participation. All participants had normal or corrected-to-normal vision, and normal color vision as determined by an Ishihara color test (described elsewhere in this article). Data from two participants (1 male) were removed from analyses owing to improper eye-tracking; all analyses thus include the remaining 28 participants.

#### Materials and stimuli

**Figure 1.**
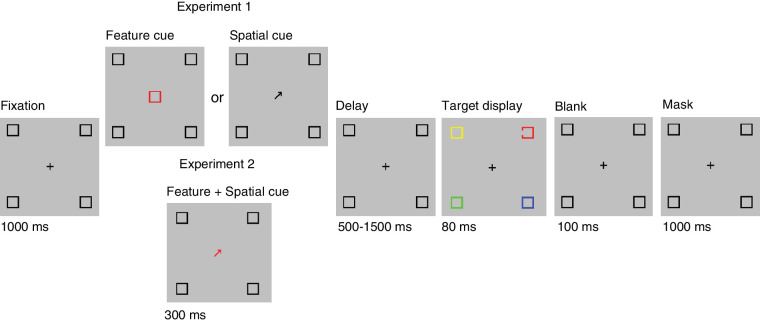
A single trial sequence in Experiment 1 (top) and Experiment 2 (bottom) with valid cues. A 300 ms central pre-cue predicted with high reliability either the color (feature cue; Experiment 1), location (spatial cue; Experiment 1), or both the color and location (feature and spatial cue; Experiment 2) of the task relevant square frame (a square with a small gap on one line segment). The target display—containing the relevant square and three unique distractor squares—appeared either 500, 1000, or 1500 ms after pre-cue offset. Participants reported the position of the small gap on the relevant square frame (left or right side). The size of the gap was adjusted throughout the experiment according to participant performance.

Stimuli were generated in MATLAB 2017b (MathWorks, Natick, MA) using Psychtoolbox3 (Kleiner et al., 2007), and displayed on a high-resolution (1920 × 1080 pixels) color monitor (BenQ XL2430T) with a frame rate of 100 Hz. Each stimulus display consisted of four uniquely colored square frames (red, yellow, blue, and green), measuring 1° width × 1° height and with a frame width of 0.1° of visual angle. The four colors used in the current study were set to be isoluminant using a color map provided by Kovesi (2015). Each square frame was positioned within a unique quadrant of the screen at a distance of 10° from central fixation. Although this is relatively far in the periphery, it is well within the 30° region in which color sensitivity is expected (Hansen et al., 2009). The target was the only square frame of the four to contain a small gap (0.25° on trial 1; see Titration Procedure) centered on either the left or right vertical edge (Figure 1). The remaining three fully closed color square frames served as distractors. The color and the position of the target and three distractors were randomly assigned on each trial.

Participants were assigned to one of two groups: spatial cue only (SC; *N* = 14) and feature cue only (FC; *N* = 14). The task paradigm was identical for both (see *Procedure*), with the only exception being the central pre-cue. The probable pre-cue for the SC group was a central black arrow, measuring 0.71° in length and 0.15° in width, pointed toward one of the four quadrants. The pre-cue for the FC group was a colored square frame the same size as the target and distractors, depicted in one of the four stimulus colors.

#### Procedure

##### Main experiment

Figure 1 illustrates the sequence of events. Participants were seated in a dark room and positioned at a distance of 92 cm from a computer monitor. Each trial began with a central black fixation cross, with each line segment measuring 1° of visual angle in length and 0.1° width. Simultaneously, four black square frames were presented in the four quadrants, matching the size and position of the colored square frames described. These squares served to highlight all four possible locations of an upcoming target. After a 1000-ms delay, the fixation cross was replaced by the 300-ms pre-cue (a black arrow for the SC group; a colored square frame for the FC group). The endogenous pre-cue accurately predicted the location (SC group) or color (FC group) of the upcoming target square frame on 80% of all trials (valid cue). For the remaining 20% of trials, the pre-cue indicated the location (SC group) or color (FC group) of a randomly selected distractor (invalid cue).

After a variable delay period of 500, 1000, or 1500 ms from cue offset, all black square frames were temporarily changed to three distractors and one target, each of which was uniquely colored red, yellow, blue, and green (see Materials and Stimuli above). The location of each object, as well as the color of the target, varied from trial to trial. All colored square frames remained on screen for 80 ms. After a 100-ms blank period, the original four black square frames reappeared on the screen to serve as backward masks, along with the fixation cross for 1000 ms. Participants were instructed to maintain fixation throughout each trial, and their compliance was verified with an eye tracker (see Eye Tracking).

Participants reported the location of the small gap on the target square frame (left or right edge) by making a speeded button press (left or right arrow key on a standard QWERTY keyboard) within a 1500-ms response window starting from mask onset. Participants completed four blocks of 150 trials each, for a total of 600 trials. The first block (structurally identical to the remaining three blocks) was treated as practice and excluded from later analysis. However, our results do not qualitatively change with its inclusion. Throughout all blocks, feedback on the accuracy of each response was given on a trial-by-trial basis. Because the task was perceptually demanding, participants were offered short breaks designated within and between blocks; otherwise, trials auto-advanced.

##### Titration procedure

Given the short stimulus exposure duration and backward masks, participants were expected to preallocate selective attention in response to the cue, in anticipation of an upcoming target after a variable delay. Maintaining top-down selective attention during relatively long delays is effortful (Ariga & Lleras, 2011; Manly, Robertson, Galloway, & Hawkins, 1999), and participants may not be inclined to do so if a target is salient enough to capture attention upon onset. Furthermore, signal enhancement tends to be weak or behaviorally undetectable via accuracy, even when selective attention is directed to a sufficiently salient target (Awh, Sgarlata, & Kliestik, 2005; Dosher & Lu, 2000; Grindley & Townsend, 1968; Scolari & Awh, 2019; Shiu & Pashler, 1994). To this end, many attention studies using similarly sparse displays have typically reported high accuracy rates for both valid and invalid cueing conditions, with attention effects restricted to RT (Egner et al., 2008; Kingstone, 1992; Lambert & Hockey, 1986). Because the goals of our study necessitated that we observe attention effects in both accuracy and RT, we implemented a titration procedure to ensure that the target gap was not salient enough to 1) capture attention in the absence of ongoing top-down deployment or 2) prevent us from observing effects of signal enhancement as a result of attentional deployment. The size of the small gap in the target square frame was titrated throughout each experimental session to achieve an overall performance criterion of 60% to 70% (similar criteria were used in Scolari & Awh (2019) and Williamson et al. (2009)). For each participant, the gap was initially set to 0.25°, or one-quarter the size of the line segment. Every 15 trials, performance accuracy was evaluated. If accuracy exceeded the upper bound of the expected range, the gap size decreased by 20% with the constraint of a 0.05° length minimum; if accuracy decreased to less than the lower bound of the expected range, the gap size increased by the same amount, with the constraint that it could not exceed 0.5°. This titration procedure was uniformly applied to both valid and invalid trials for the course of the full experiment to allow for possible deviations in attentional control over time (Manly et al., 1999). Importantly, the overall mean accuracy should fall within the performance criterion range for each participant, but without artificially restricting how much performance in any given condition could deviate from the mean.

##### Ishihara color test

Because color was a critical feature in the main experiment, participants first completed a computerized Ishihara color test (Nakajima, Ichikawa, Nakagawa, Majima, & Watanabe, 1960) to ensure all had normal color vision. Each participant reported, orally and without time pressure, what number or pattern was embedded in a color dot pattern on the screen (if any). All participants passed this screening, and these data were discarded from further analyses.

##### Eye tracking

Each participant's right eye gaze position was recorded at a sampling rate of 500 Hz via an Eyelink 1000 Plus system (SR Research, Ontario, Canada) for the full length of each trial. The eye-tracking camera was positioned in front of the stimulus presentation monitor, approximately 55 cm from the participant. The tracker was calibrated for each participant's eye using 13 reference points in the chinrest-free mode before the start of the experiment. For the purposes of data analysis, an interest time period was set from pre-cue onset to target offset. An interest area was defined as a square of 3.33° × 3.33° (less than one-half of the distance – 4.7°, between fixation and any one of the possible target positions) centered on fixation. Individual trials were removed from further analyses offline if the participant blinked or made a saccade outside of the interest area during the interest period.

#### Data analysis

##### Attention effects in accuracy and RT

We examined participants’ performance via accuracy (d′) and RT for each pre-cue group. First, trials in which responses were made in 250 ms or less were removed for all analyses (Wagenmakers et al., 2007) to eliminate what are likely to be fast guesses. RT analyses were further restricted to correct trials only. Next, we calculated attention effects by subtracting the invalid pre-cue condition from the valid pre-cue condition in both accuracy and RT. Because participants were randomly assigned to each of the two pre-cue groups, a 2 × 2 (pre-cue group x cue validity) mixed-design analysis of variance (ANOVA) was deployed to analyze the results.

##### Titration outcomes

As described in *Titration procedure*, we used a staircasing procedure to titrate the size of the target gap with the goal of matching task difficulty across participants (see *Titration procedure*). In addition to compensating for individual differences in perceptual ability, the outcome of this procedure can serve as an objective measure of the relative effectiveness of the endogenous pre-cues. If both the spatial and feature pre-cues are equally effective at facilitating target processing on the valid trials, then we should observe no differences in gap size between groups. First, we verified that the average gap size between validly and invalidly pre-cued trials was matched for both SC and FC groups using a pair of within-participants *t* tests, so that no external saliency differences between pre-cueing conditions could account for any observed attention effects. Then, we conducted a between-participants comparison to determine whether the average gap size across the length of the experiment differed between groups.

##### Robust EZ-diffusion model

We used a two-alternative forced choice task where participants were instructed to respond as accurately and as quickly as possible to a target, allowing us to examine accuracy and RT across conditions. Separately, these two measurements can show us how fast and how well participants localized the gap in the target square frame. However, considering their weighted contributions together to form a unified behavioral response provides us with a more nuanced view of these data. We therefore used a robust EZ-diffusion model.

The EZ-diffusion model is a simple modification of the Ratcliff diffusion model (Ratcliff, 1978; Ratcliff & McKoon, 2008) that estimates three critical components to decision making: 1) drift rate (*v*), or the rate at which evidence is accumulated for a given response alternative, 2) boundary separation (*a*), or the conservativeness of a response criterion, and 3) non-decision time (*T_er_*), or the proportion of time spent outside of evidence accumulation, including some perceptual and motor processes (see Wagenmakers et al., 2007; Wagenmakers et al., 2008). The robust EZ, an extension to the original, additionally models out contaminated data that may result from lapses of attention and thus add noise and/or inaccuracies to the aforementioned estimates (Ratcliff, 2008; Wagenmakers et al., 2008).

To estimate these parameters, three key measurements from each participant and each condition are entered into the model: proportion of correct responses (*P_c_*), mean correct RT (*MRT*), and the variance of the correct RT (*VRT*). At the same time, three important assumptions must be made: 1) the RT data are skewed rightward to some degree; 2) the RT distributions for correct and incorrect responses are identical; and 3) the starting point (before evidence accumulation) is equidistant from both response alternative decision boundaries. The model has been shown to perform well when small violations to these assumptions are present (Wagenmakers et al., 2007), but the output should be interpreted with caution in the case of severe violations. Thus, we report the results of our misspecification checks in the Results and Discussion.

### Results and discussion

The goal of Experiment 1 was to demonstrate that both the feature and spatial cues were independently effective at directing attention to the target and facilitating perceptual decision making. Although we used three cue-to-target interstimulus intervals in our design (500, 1000, and 1500 ms; see Procedure), we did not observe reliable interactions with interstimulus intervals in any comparisons. Thus, we collapse across all delays in the following analyses.

To accurately assess attention effects in our sparse display, it was critical that participants maintain fixation from cue onset to response. Thus, data were first preprocessed by removing trials in which participants blinked or made saccades and/or fixations outside of a central interest area (see Eye Tracking). As a result, 13.07% of trials were removed on average across participants. Next, trials in which button presses were made in less than 250 ms, signaling that the participant was making a fast guess, were removed from analyses (7.5% of trials on average), as well as trials in which neither of two response buttons were selected (1.01% of trials on average). All analyses reported below were conducted after these preprocessing steps.

#### Attention effects in accuracy and RT

**Figure 2.**
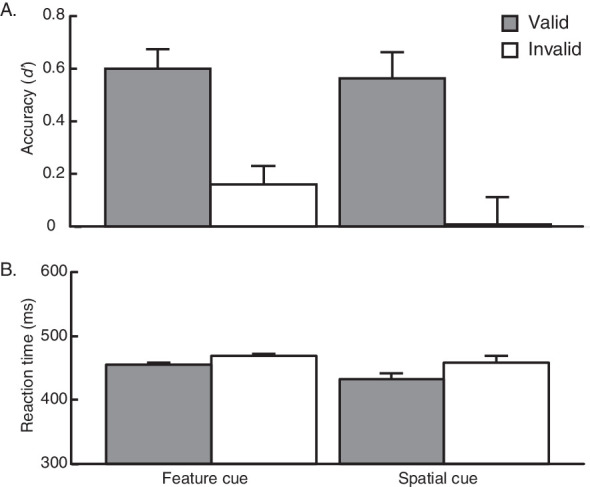
Results from Experiment 1, where participants were either presented with a feature cue (FC group) or a spatial cue (SC group). Performance accuracy (A) and reaction time (B) on valid and invalid trials are plotted for each of the two groups. Mean reaction times (RTs) were calculated for correct trials only. Error bars reflect ±1 within-participant SEM.

Across both groups, participants appropriately used the endogenous pre-cue to attend to the target, as indicated by behavioral performance: the valid cue led to better performance than the invalid cue in both accuracy (*d′* (percent correct in parentheses) for valid: *M* = 0.58 (60.9%); invalid *M* = 0.084 (52%)) and RT (valid *M* = 443.5 ms; invalid *M* = 464 ms). A 2 × 2 (pre-cue group x cue validity) mixed-design ANOVA revealed that these differences were significant (accuracy: *F*(1, 26) = 31.13, *p* < 0.001, $\eta_{G}^{2}$ = 0.33; see Figure 2A; RT: *F*(1,26) = 7.69, *p* = 0.01, $\eta_{G}^{2}$ = 0.03; see Figure 2B).

Critically, both groups performed equally well on the task, *F*(1, 26) = 0.81, *p* = 0.38, $\eta_{G}^{2}$ = 0.02, and we did not observe a significant pre-cue group by cue validity interaction within accuracy, *F*(1, 26) = 0.39, *p* = 0.54, $\eta_{G}^{2}$ = 0.006. Furthermore, a pair of within-participants *t* tests revealed that both groups exhibited significant attention effects in accuracy: FC group, *t*(13) = 4.29, *p* = 0.00088, *d* = 1.15; SC group, *t*(13) = 3.80, *p* = 0.0022, *d* = 1.02. Similarly, RTs were statistically matched between groups, *F*(1, 26) = 0.64, *p* = 0.43, $\eta_{G}^{2}$ = 0.02, and there was no significant interaction between factors, *F*(1,26) = 0.92, *p* = 0.35, $\eta_{G}^{2}$ = 0.004. Within-participants *t* tests further revealed that attention effects in RT were significant for the FC group, *t*(13) = 2.62, *p* = 0.02, *d* = 0.70, and marginal in the SC group, *t*(13) = 1.99, *p* = 0.068, *d* = 0.53. Thus, both the spatial and feature pre-cues elicited attention effects of similar magnitudes.

#### Titration outcomes

**Figure 3.**
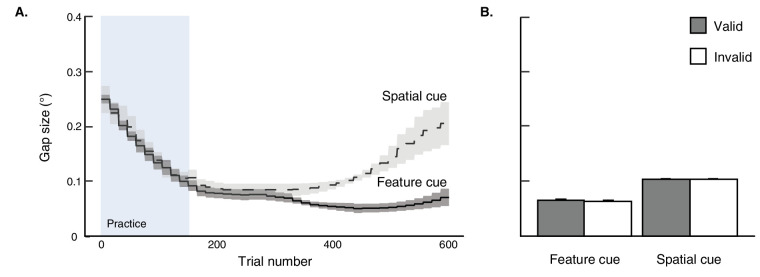
Target gap size (in degrees) from Experiment 1 are plotted for the FC and SC groups, respectively, on a trial-by-trial basis (A) and averaged over valid and invalid trials for each group outside of the practice block (B). Error bars reflect ±1 within-participant SEM.

We titrated the size of the gap on the target square frame to ensure that task difficulty was matched across participants. The gap size generally reached asymptotic levels for most participants by the end of the practice block, and did not significantly differ between valid and invalid trials over the course of the experiment: FC group, *t*(13) = 1.27, *p* = 0.227, *d* = 0.029; SC group, *t*(13) = 0.049, *p* = 0.962, *d* = 0.0008 (Figures 3A and 3B).

To the extent that the spatial and feature cues are equally effective in both guiding selective attention and resolving a perceptually demanding target, each should produce equivalently small gap sizes across participants. Although the mean gap size was qualitatively larger for the SC group, an unequal variances two-sample *t* test showed there was no significant difference between groups, *t*(14.28) = 1.22, *p* = 0.244, *d* = 0.46. This finding suggests that both cue types were equally effective in facilitating target identification.

#### Robust EZ-diffusion model

To further investigate how use of the pre-cue informed perceptual decision making, we next used the robust EZ-diffusion model (Wagenmakers et al., 2008). First, we checked that the assumptions of the model were met. Across participants, the distributions of RTs for both correct and incorrect trials are right skewed (FC group: correct, skew = 1.26; incorrect, skew = 1.02; SC group: correct, skew = 1.36; incorrect, skew = 1.11, where a nonzero positive value indicates rightward skew; see Supplementary Figure S1 for individual participant plots). We next conducted a series of within-participant *t* tests to determine whether the RT distribution for correct and incorrect responses were statistically matched for each participant (Wagenmakers et al., 2007; see Supplementary Figure S1). Using uncorrected *p* values, 6 of the 28 participants (21.4%) violated this assumption (smallest *p* = 0.004). This finding is approximately on par with the percentage of participants who violated the same assumption in the original application of this model and were nonetheless included in the full analyses (18%; Wagenmakers et al., 2007). When our series of *p* values were subjected to a false discovery rate (FDR) correction (Benjamini & Hochberg, 1995), none of the significant values remained (range of *p* = 0.11 to *p* = 0.99; mean *p* = 0.47). Thus, the first two assumptions of the model are satisfactorily met.

Next, we set out to determine whether participants exhibited a starting point bias toward one response alternative over another. First, we found that, on average, participants selected each response alternative (leftward gap versus rightward gap) at a similar frequency, *t*(27) = 1.73, *p* = 0.096, *d* = 0.33. We then conducted a series of two-way ANOVAs—one for each participant—investigating whether individual participants showed an RT bias toward one alternative over the other (i.e., by making comparatively faster correct responses to one alternative and comparatively slower correct responses to the other, as would be demonstrated by a crossover interaction between stimulus alternative and response accuracy; Wagenmakers et al., 2007; see Supplementary Figure S2). Of the 28 participants, eight exhibited a significant crossover interaction (28.6%; smallest *p* = 0.0001), suggesting a starting point bias for those individuals (well-matched with the 28.9% included in the model who showed a similar bias in Wagenmakers et al., 2007). After FDR correction, only four participants maintained evidence of a bias (range of *p* = 0.0027 to *p* = 0.99; mean *p* = 0.42).

**Figure 4.**
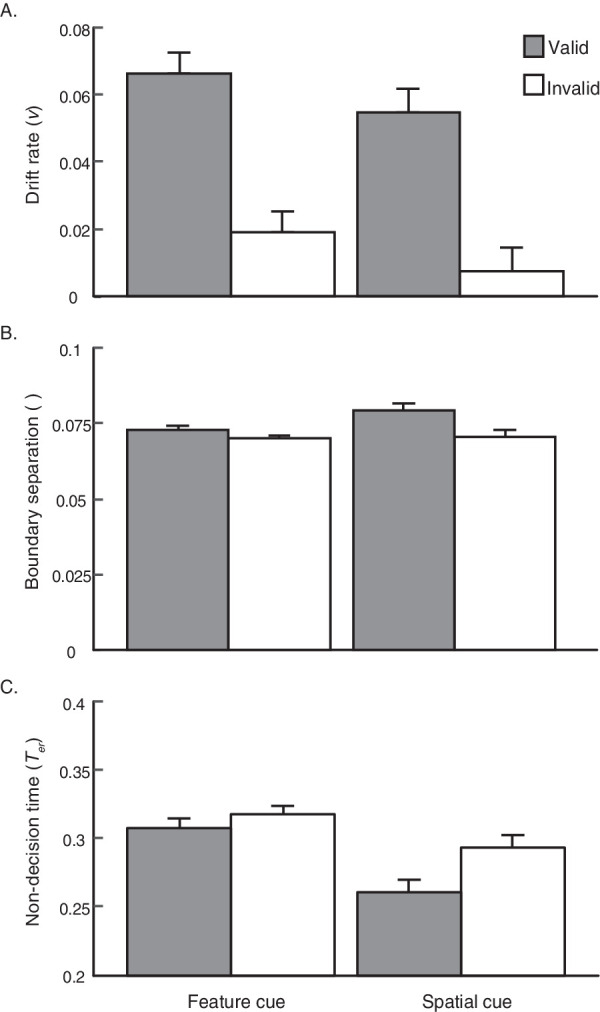
Results from the robust EZ-diffusion model are plotted for valid and invalid trials from the FC and SC groups from Experiment 1. Drift rate (A) indicates the speed of evidence accumulation; boundary separation (B) indicates response conservativeness; and non-decision time (C) indicates the time interval that occurs outside of evidence accumulation. Error bars reflect ±1 within-participant SEM.

To check the robustness of the model against these individual instances of violated assumptions, we removed the four participants who exhibited a starting point bias (see Supplementary Material for Experiment 1 and Supplementary Figure S3). The patterns of results across all model outputs were qualitatively matched regardless of whether they were included. We thus report the three parameter estimates, including drift rate, boundary separation, and non-decision time in turn below, with all participants included (Figure 4).

##### Drift rate

The drift rate parameter serves as a measure of the speed at which evidence in favor of one response alternative over the other is accumulated. Recall that, in this task, each participant covertly attended to the relevant square frame to determine whether the target gap was present on the left or right vertical edge; thus, the response alternatives are left or right.

We reasoned that a sharper perceptual representation of the relevant item should result in faster evidence accumulation; thus, the drift rate can serve as an indirect indicator of signal enhancement, much like performance accuracy. As would be expected, the drift rate was greater following a valid pre-cue (*M* = 0.06) than after an invalid pre-cue (*M* = 0.013) across both cue types, *F*(1, 26) = 23.10, *p* < 0.001, $\eta_{G}^{2}$ = 0.26; Figure 4A. Both groups showed this pattern: FC group, *t*(13) = 3.65, *p* = 0.0029, *d* = 0.98; SC group, *t*(13) = 3.19, *p* = 0.0071, *d* = 0.85, and drift rate estimates did not differ between groups, *F*(1, 26) = 0.95, *p* = 0.34, $\eta_{G}^{2}$ = 0.02. We also did not observe a significant interaction between groups and cue validity within drift rate, *F*(1, 26) = 0.02, *p* > 0.99, $\eta_{G}^{2}$ < 0.0001. Thus, both the valid spatial and valid feature cues elicited equivalent rates of evidence accumulation.

##### Boundary separation

The boundary separation parameter serves as a measure of response conservativeness, or the relative amount of acquired discriminative evidence that precedes response execution. Greater response caution suggests slower reaction times with typically fewer errors; relatively decreased response caution suggests faster responses at the risk of more errors (i.e., a speed–accuracy trade-off). Boundary separation was numerically larger on valid trials (*M* = 0.076) compared with invalid trials (*M* = 0.070), and the difference was statistically significant across groups, *F*(1, 26) = 5.38, p = 0.03, $\eta_{G}^{2}$ = 0.07. However, this pattern did not emerge when considering each individual group alone, nonsignificant for the FC group, *t*(13) = 1.21, *p* = 0.25, *d* = 0.32; marginal for the SC group, *t*(13) = 1.98, *p* = 0.069, *d* = 0.53 (Figure 4B). Boundary separation estimates were not significantly different between groups, *F*(1, 26) = 1.27, *p* = 0.27, $\eta_{G}^{2}$ = 0.03, and, although the difference between validity conditions was numerically larger for the SC group, there was no reliable interaction between pre-cue group and validity, *F*(1, 26) = 1.30, *p =* 0.27, $\eta_{G}^{2}$ = 0.02. These results suggest, albeit somewhat weakly, that participants accumulated more evidence before response selection after a valid pre-cue than an invalid pre-cue, thus resulting in relatively more conservative responses.

##### Non-decision time

Non-decision time refers to the proportion of RT spent outside of evidence accumulation (as captured by drift rate and boundary separation). In our paradigm, this would include verifying that attention had been preallocated to the correct item, and, in the event of an invalid cue, potentially making additional attentional shift(s). Note that the 80-ms target duration should preclude volitional shifts of attention during stimulus presentation (Itti & Koch, 2001; Liu, Stevens, & Carrasco, 2007), in which case any shift would occur among internal representations of the stimuli from which to accumulate evidence. Non-decision time additionally includes motor response execution once a decision criterion is reached (Wagenmakers et al., 2007); however, because the speed of the motor response is not expected to vary with cue validity, we focus on early components of non-decision time when interpreting any cueing effects.

Echoing drift rate and boundary separation estimates, we observed a significant cue validity effect in non-decision time collapsed across groups, *F*(1, 26) = 6.63, *p* = 0.02, $\eta_{G}^{2}$ = 0.07; see Figure 4C. Participants generally spent less time preparing for evidence accumulation following a valid cue (*M* = 0.28) than an invalid cue (*M* = 0.31). Departing from the previous two parameter estimates, however, here we observed a significant pre-cue group effect, *F*(1, 26) = 7.32, *p* = 0.01, $\eta_{G}^{2}$ = 0.17: across valid and invalid trials, spatial cues resulted in shorter non-decision times (*M* = 0.28) than feature cues (*M* = 0.31). This result may be related to previous research findings that FBA is slower to activate than SBA (Anllo-Vento & Hillyard, 1996; Liu, Stevens, & Carrasco, 2007). Furthermore, a pair of within-participant *t* tests indicate that the validity effect reported above was restricted to the SC group (SC: *t*(13) = 2.38, *p* = 0.033, *d* = 0.64; FC: *t*(13) = 1.065, *p* = 0.31, *d* = 0.28), but an interaction between pre-cue group and validity failed to reach significance, *F*(1, 26) = 1.95, *p* = 0.17, $\eta_{G}^{2}$ = 0.02.

The attention effect observed for the SC group may not be surprising, given an invalid spatial cue may require a shift between internal object representations before the target gap can be localized, while a similar shift is unnecessary in response to a valid cue. Because internal representations generated from unattended stimuli are more degraded than those from attended stimuli (Carrasco, 2006; 2011; Liu et al., 2009), a shift to the former for the purpose of evidence accumulation should additionally produce a slower drift rate (as reported in *Robust EZ-diffusion model: Drift rate*).

We do not have a firm explanation at this time as to why a valid feature cue would not similarly reduce non-decision time. To speculate, though, we consider the global nature of FBA: attention is deployed to sensory populations across the full visual field that respond maximally to the cued feature, regardless of the (anticipated) target location (Rossi & Paradiso, 1995; Serences & Boynton, 2007; White & Carrasco, 2011). Given that our task was to detect a single target gap that was purposefully orthogonal to the attended color, only one subset of the activated sensory populations is informative; the others contribute noise. Thus, identifying the most informative signal may be necessary on both valid and invalid trials before evidence accumulation can begin (this explanation would similarly be consistent with the overall longer non-decision time for the FC group). Even so, we would still expect a slower drift rate to result on invalid trials (again, as reported in *Robust EZ-diffusion model: Drift rate*) because the color of the relevant square was misaligned with the cue and therefore unattended, thus producing a noisier representation of the relevant item. Note that, if this explanation is correct, it would hold for numerous existing FBA studies in which the attended feature is used to identify an orthogonal target (e.g., Liu et al., 2007; Serences & Boynton, 2007; White et al., 2015).

In this experiment, perceptual decision making was facilitated by both the spatial and feature cues. Given statistically equivalent attention effects in accuracy and RT, as well as equivalent average gap sizes, we conclude that both cue types were equally effective in directing attention and resolving the perceptually challenging target. Importantly, patterns across decision-making components estimated by the diffusion model suggest some differences between selection mechanisms. Although attention effects within RT were matched, our model outputs suggest divergent accounts for those effects: Given that a feature cueing effect was only reliably observed in drift rate, FBA effects in RT may be restricted to evidence accumulation. In contrast, a spatial cueing effect was reliably observed in both drift rate and non-decision time, such that a valid spatial cue both speeded the onset of evidence accumulation and accelerated the rate at which it occurred. We tentatively account for these differences by invoking the unique ways in which space-based and feature-based modulatory signals are distributed across sensory populations according to their spatial and color selectivity, respectively. Despite these differences, our conclusions with respect to the effectiveness of each cue type remain.

Having established the equivalent effectiveness of a lone spatial and lone feature cue in enhancing the representation of a perceptually challenging target, we next turned to our main objective: to investigate whether and how SBA and FBA interact to facilitate target identification in a sparse display.

## Experiment 2

Experiment 1 demonstrated that our design is sufficient to elicit comparable SBA and FBA effects in accuracy, RT, and rates of evidence accumulation. The larger goal of this study, however, is to explore if and how these two selection mechanisms interact to facilitate perceptual decision making of a single item when both space and feature information is made available simultaneously via an endogenous pre-cue. We used the same task paradigm from Experiment 1, changing only the pre-cue: here, we used a colored arrow that indicated both the color and location of an upcoming target with equal probability (80%). If SBA and FBA are deployed independently, we should observe the same pattern of results reported in Experiment 1 for each cue type, and an additive effect when both are valid. If, however, the two mechanisms interact, we should expect a different pattern that may reveal the nature of this interaction.

### Methods

#### Participants

Thirty-one undergraduate students (2 males) with normal or corrected-to-normal vision were recruited through the Texas Tech University research participation system (SONA) and received course credit for their participation. A target sample size of 30 participants was selected a priori to roughly match the sample size in Experiment 1. All participants gave written informed consent as required by the institutional review board and in compliance with the Declaration of Helsinki. Each had normal color vision as determined by the Ishihara color test (see Experiment 1 Methods). One participant withdrew before completing the task, and hence was excluded from the analyses; the remaining 30 participants are included in all analyses.

#### Materials and stimuli

The material and stimuli matched those reported in Experiment 1.

#### Procedure

##### Main experiment

The procedure of the main experiment largely matched those reported in Experiment 1, except here the independent space and feature pre-cues were combined in a single colored arrow (Figure 1). The validity of the pre-cue was as follows: for 70% of the trials, it accurately predicted both the spatial location and color of the upcoming target square frame (space- and feature-valid condition, or wholly valid); for 10% of the trials, it accurately predicted only the location of the target square frame (space valid/feature invalid); for 10% of the trials, it accurately predicted only the color of the target square frame (feature valid/space invalid); and for the remaining 10% of the trials, it predicted neither the location nor the color of the upcoming target square frame (wholly invalid). Notably, this means that location and color were each validly cued on 80% of all trials (consistent with Experiment 1), and that a strategy that used both selection mechanisms independently would result in the greatest performance accuracy.

Participants completed a total of 1,050 trials over the course of seven blocks. The first block of 150 trials was treated as practice and excluded from later analysis.

##### Titration procedure

See Experiment 1 Methods for a description of the titration procedure used here.

##### Ishihara color task

See Experiment 1 Methods for a description of the Ishihara color test.

##### Eye tracking

See Experiment 1 Methods for a description of the eye tracking apparatus and procedures.

#### Data analysis

##### Attention effects in accuracy and RT

A 2 × 2 (spatial cue validity × feature cue validity) repeated-measures ANOVA was deployed to examine participants’ performance via accuracy (*d′*) and correct RTs. Attention effects in both measures were calculated by subtracting the relevant invalid pre-cue from the relevant valid pre-cue, while holding the validity of the remaining cued dimension constant. For example, the SBA effect on feature-invalid trials was calculated as space valid minus wholly invalid, and on feature-valid trials, as wholly valid minus feature valid. The same logic was applied to FBA effects.

##### Titration outcomes

Just as we did in Experiment 1, we used a staircasing procedure to titrate the size of the target gap on a participant-by-participant basis. We again used the outcome of this procedure as an objective measure of the relative effectiveness of the endogenous pre-cues. In this case, all participants were exposed to the same pre-cues, and all pre-cues for each participant were included in a single staircase. Thus, here we divided participants into one of four groups based on their pre-cue preference. To do this, we averaged across the magnitude of each participants’ attention effects in accuracy when 1) the remaining pre-cue component was invalid and 2) the remaining pre-cue component was valid, and plotted the results in an orthogonal, two-dimensional space. We then used this visualization to classify participants as exhibiting a preference for the spatial cue, feature cue, both, or neither. Finally, we made between-participant comparisons of average gap size across the length of the task. This analysis allowed us to investigate whether individual differences in pre-cue preference produced different degrees of perceptual facilitation.

##### Robust EZ-diffusion model

The robust EZ-diffusion model was again used using the same analysis procedure as described in Experiment 1, following our misspecification checks.

### Results and discussion

The goal of Experiment 2 was to investigate if and how SBA and FBA interact to facilitate perceptual decision making of a single, non-salient target. As in Experiment 1, we did not observe any reliable interactions with the three interstimulus intervals used in our design (500, 1000, and 1500 ms; see Experiment 1 Procedure), and so we collapse across delays in the following analyses.

The same preprocessing steps described in Experiment 1 were used here, before the data analyses described next. On average, 13.7% of trials were removed due to blinks or saccades away from fixation; 9.75% of trials were removed because response times occurred in or under 250 ms; and another 2.1% of trials were removed because neither of the two response keys were selected.

#### Attention effects in accuracy and RT

**Figure 5.**
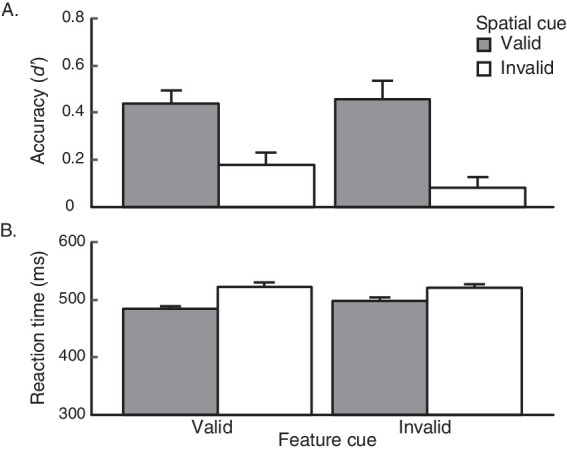
Results from Experiment 2, where all participants were presented with an integrated space and feature cue. Performance accuracy (A) and reaction time (B) are plotted for space-valid, feature-valid, space- and feature-valid, and invalid trials. Mean reaction times (RTs) were calculated for correct trials only. Error bars reflect ±1 within-participant SEM.

Figure 5A depicts the average accuracy for each trial type (wholly valid, feature valid, space valid, wholly invalid). We observed a significant space-based cueing effect: *d′* (percent correct in parentheses) for valid: *M* = 0.44 (58.2%); invalid: *M* = 0.13 (52.4%), *F*(1,29) = 10.70, *p* = 0.003, $\eta_{G}^{2}$ = 0.1. No such cueing effect was observed in response to the feature cue: *d′* (percent correct in parentheses) for valid: *M* = 0.40 (57.6%); invalid: *M* = 0.27 (54.8%), *F*(1,29) = 0.47, *p* = 0.50, $\eta_{G}^{2}$ = 0.002. Furthermore, we did not observe an interaction between cue types, *F*(1,29) = 0.82, *p* = 0.37,$\;\eta_{G}^{2}$ = 0.004. A series of FDR-corrected *t* tests confirmed that the SBA effect was significant for feature-valid trials, *t*(29) = 2.42, *p* = 0.044, *d* = 0.44, and feature-invalid trials *t*(29) = 3.11, *p* = 0.017, *d* = 0.57. Thus, the size of the SBA effect was not modulated by the validity of the feature cue. At the same time, there was no detectable FBA effect regardless of location validity: space-valid, *t*(29) = -0.26, *p* = 0.79, *d* = -0.048; space-invalid, *t*(29) = 1.06, *p* = 0.40, *d* = 0.19.

This somewhat surprising albeit straightforward pattern of results indicates that participants did not use the feature cue to identify the target, despite its expected equivalent reliability and independent effectiveness with the spatial cue (as demonstrated in Experiment 1). The mean accuracy was admittedly low for a two-alternative forced choice task at 57.02%, such that task difficulty could have had unintended consequences on participants’ willingness to effortfully deploy top-down attention. Notably, however, the mean accuracy was similar in Experiment 1 (59.35%), where we observed both effects separately. Furthermore, when we considered only the first two blocks of Experiment 2 (including the practice block), where the mean accuracy was higher at 65.36% and each of the three valid cue types was significantly above chance, we observed the same pattern of results: significant SBA effect, *F*(1, 29) = 20.36, *p* < 0.0001, $\eta_{G}^{2}\;$= 0.25; nonsignificant FBA effect, *F*(1, 29) = 0.83, *p* = 0.37, $\eta_{G}^{2}$ = 0.004; no interaction, *F*(1, 29) = 0.08, *p* = 0.78, $\eta_{G}^{2}$ = 0.0004. Therefore, we conclude that participants relied solely on SBA for target identification.

Just as we observed in accuracy, a 2 × 2 ANOVA revealed a significant space-based cueing effect in RT, *F*(1, 29) = 18.78, *p* < 0.001, $\eta_{G}^{2}$ = 0.05, but not a feature-based cueing effect *F*(1,29) = 1.86, *p* = 0.18, $\eta_{G}^{2}$ = 0.002; see Figure 5B. A series of FDR-corrected *t*-tests confirmed that the SBA effect was significant for both feature-valid, *t*(29) = 4.28, *p* = 0.0047, *d* = 0.78, and feature-invalid trials, *t*(29) = 2.51, *p* = 0.036, *d* = 0.46. There was no detectable FBA effect on space-invalid trials, *t*(29) = -0.35, *p* = 0.73, *d* = 0.064, and although one did appear to emerge on space-valid trials, *t*(29) = 2.89, *p* = 0.0097, *d* = 0.53, an interaction between cue types did not reach significance, *F*(1,29) = 2.69, *p* = 0.11, $\eta_{G}^{2}$ = 0.005. We revisit this pattern in the *EZ-Diffusion Model* section below.

#### Titration outcomes

Because we observed FBA effects in accuracy when the feature cue was presented alone in Experiment 1, we admittedly expected to find evidence of feature cue use in target identification here as well. Across all participants, the data demonstrate clearly that this was not the case. Notably, however, we observed individual differences in cueing effects, such that some participants exhibited only an SBA effect in accuracy, whereas others exhibited both SBA and FBA effects. It is possible, then, that those who showed both cueing effects in accuracy used the two selection mechanisms to resolve the target. We thus divided our participants into groups according to whether they exhibited qualitative FBA, SBA, or SBA and FBA effects. Following similar logic given in Experiment 1, if SBA and FBA can be combined to improve perceptual resolution—either additively or super-additively—we would expect significantly smaller gap sizes for the participants who showed attention effects for both cue types.

**Figure 6.**
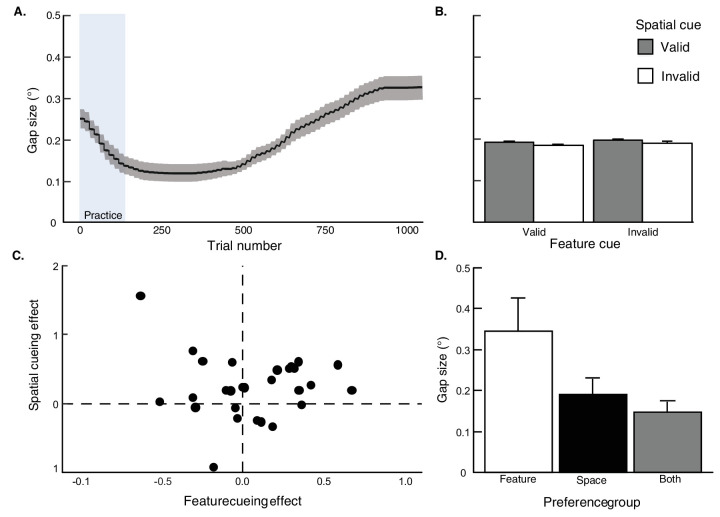
Target gap sizes (in degrees) from Experiment 2, depicted on a trial-by-trial basis (A) and averaged over valid and invalid trials for both the feature and spatial cues outside of the practice block (B). Participants were assigned to cue preference groups based on their cueing effects in accuracy (d′). Participants’ accuracy scores are plotted in a four-quadrant space (C), where the feature cueing effect is defined as feature-valid–invalid performance, and the spatial cueing effect is defined as space-valid–invalid performance. Each dot is a single participant. Participants with a positive feature cueing effect (only) were assigned to the feature preference group (*n* = 3); those with a positive spatial cueing effect (only) were assigned to the space preference group (*n* = 11); those with both positive feature and space cueing effects were assigned to the both preference group (*n* = 12). The target gap size (D) is plotted for each preference group. Error bars reflect ±1 within-participant SEM.

First, visual inspection of the trial-by-trial average gap size revealed that asymptote was reached after the practice block and remained largely stable for the first 500 trials, echoing Experiment 1. However, it steadily increased again after this point, perhaps due to participant fatigue (note that Experiment 2 had 75% more trials than Experiment 1). Importantly, gap size stabilized again for approximately the last 100 trials and remained below the upper-bound (see Figure 6A). Furthermore, average gap size across participants was similar for all four trial types, *F*(3, 87) = 1.99, p = 0.12, $\eta_{G}^{2}$ < 0.001 (see Figure 6B).

We next visualized participants’ performance in a four-quadrant space, where the *x*-axis demarks the feature cue effect on accuracy, and the y-axis demarks the orthogonal spatial cue effect (Figure 6C). Each quadrant represents a unique cue preference: spatial cue (*n* = 11), feature cue (*n* = 3), both spatial and feature cues (*n* = 12), and neither cue (*n* = 4). Given the small number of participants who showed a feature cue-only preference, we elected to only compare the two preference groups that either showed reliance on the spatial cue or both spatial and feature cues. If using both cues results in greater perceptual resolution, we would expect our titration procedure to produce on average smaller gap sizes for the latter group. However, this was not the case: gap sizes were matched between groups, *t*(22) = 0.86, *p* = 0.40, *d* = 0.37 (Figure 6D). This finding suggests that, even when participants used the feature cue in conjunction with the spatial cue to identify the target, it did not seem to improve their perceptual resolution of the target gap.

#### Robust EZ-diffusion model

The performance accuracy and gap size results described suggest that, across participants, SBA alone contributed to the perceptual resolution of the target. At the same time, the RT data trended toward an (albeit insignificant) interaction between cue types, with an FBA effect emerging only on space-valid trials. Notably, RT combines distinct elements of decision making, such as signal strength, response conservativeness, and processing speed (Ratcliffe, 1978; Wagenmakers et al., 2007; White et al., 2015). Thus, although an interaction between cue types did not reach significance when considering RT as a unitary measure, we nonetheless may observe reliable interactions for distinct portions of response time. We therefore next used the EZ-diffusion model to further investigate the contributions of each selection mechanism during unique components of perceptual decision making.

As in Experiment 1, we first verified that all three assumptions of the model were met. The average distributions of RT for correct and incorrect trials were both right skewed as expected (correct: skew = 0.89; incorrect: skew = 0.73; see Supplementary Figure S4 for individual participant plots), satisfying the first assumption. We also determined that correct and incorrect RT distributions were largely overlapping for the majority of participants, although eight of the 30 participants violated this assumption based on uncorrected *p*-values (smallest *p* < 0.001); this rate is decreased to seven of 30 after FDR correction (23.3%; range of *p* < 0.001 to *p* < 0.99; mean *p* = 0.43; see Supplementary Figure S4). Again, this is on par with the violation rate reported in Wagenmakers et al. (2007). Finally, we looked to see whether participants exhibited a starting point bias, in violation of the third assumption. On average, there was no bias toward one response alternative over another, *t*(29) = 0.27, *p* = 0.79, *d* = 0.05. However, a series of two-way ANOVAs revealed that nine of the 30 participants exhibited a starting point bias as depicted by a significant crossover interaction (decreased to eight of 30 after FDR correction; Supplementary Figure S5), which is consistent with the rate reported in Wagenmakers et al. (2007).

**Figure 7.**
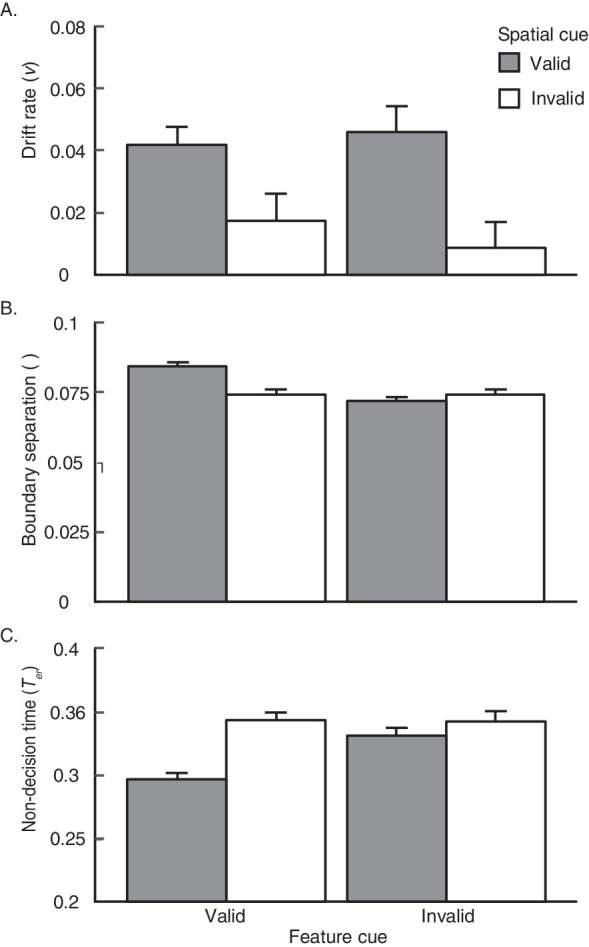
Results from the robust EZ-diffusion model are plotted for all participants from Experiment 2. Drift rate (A), boundary separation (B), and non-decision time (C) are plotted for space-valid, feature-valid, space- and feature-valid, and invalid trials. Error bars reflect ±1 within-participant SEM.

As in Experiment 1, we checked the robustness of the model against individual instances of violated assumptions by removing the participants who exhibited 1) a significant difference in RT between correct and incorrect trials, and/or 2) a starting point bias (12 participants total; see Supplementary Material for Experiment 2 and Supplementary Figure S6). Although the smaller sample size affected our power to detect significance in some cases, the patterns across model outputs were largely matched regardless of whether they were included. We thus report the three parameter estimates, including drift rate, boundary separation and non-decision time in turn below, with all participants included (Figure 7).

##### Drift rate

The drift rate estimates of the EZ-diffusion model mirrored the results we observed in accuracy (Figure 7A). The drift rate was significantly faster after a valid spatial cue compared with an invalid spatial cue, *F*(1, 29) = 9.39, *p* = 0.005, $\eta_{G}^{2}$ = 0.09. This pattern was marginally significant on feature-valid trials, *t*(29) = 2.27, *p* = 0.062, *d* = 0.41, and significant on feature-invalid trials, *t*(29) = 2.83, *p* = 0.034, *d* = 0.52. No such cueing effect was observed for the feature cue, *F*(1, 29) = 0.14, *p* = 0.72, $\eta_{G}^{2}$ = 0.0006, regardless of the validity of the spatial cue: space-invalid, *t*(29) = 0.83, *p* = 0.55, *d* = 0.15; space-valid, *t*(29) = −0.53, *p* = 0.60, *d* = −0.10. Furthermore, we did not observe an interaction between pre-cue types, *F*(1, 29) = 0.98, *p* = 0.33, $\eta_{G}^{2}$ = 0.004, suggesting that the size of the SBA effect did not change with the inclusion of a valid feature pre-cue. We, therefore, conclude that SBA alone contributed to the rate of evidence accumulation, much the same as we observed with accuracy.

##### Boundary separation

Unlike *d′* and drift rate—measures we interpret as indicative of attention-related perceptual enhancements—we observed influences of both pre-cue types in response caution. When collapsed across valid and invalid spatial pre-cues, valid feature pre-cues were followed by more conservative responses than invalid feature pre-cues, *F*(1, 29) = 16.45, *p* = 0.0003, $\eta_{G}^{2}$ = 0.08. The same validity effect was true for spatial pre-cues as well, *F*(1, 29) = 11.54, *p* = 0.002, $\eta_{G}^{2}$ = 0.03. For both cue types, the validity effect appears to be largely driven by the wholly valid condition (Figure 7B), and indeed, we observed a significant interaction between pre-cue types, *F*(1, 29) = 13.45, *p* = 0.001, $\eta_{G}^{2}$ = 0.09. A series of FDR corrected *t* tests confirmed that cueing effects were present only when both cue types were valid: SBA effect, feature-valid, *t*(29) = 5.20, *p* < 0.001, *d* = 0.95; feature-invalid, *t*(29) = 1.14, *p* = 0.35, *d* = 0.21; FBA effect, space-valid, *t*(29) = 6.90, *p* < 0.001, *d* = 1.26; space-invalid, *t*(29) = 0.087, *p* = 0.93, *d* = 0.016. Thus, participants accumulated relatively more evidence before executing a response on trials in which both the spatial and feature pre-cues were valid.

##### Non-decision time

 Figure 7C depicts the non-decision time for each pre-cue type. Like boundary separation, we again found evidence of influence from both the feature and spatial cues. Non-decision time was significantly shorter following a valid feature cue compared with an invalid feature cue, *F*(1, 29) = 8.12, *p* = 0.008, $\eta_{G}^{2}$ = 0.02. The same validity effect was observed for space, *F*(1, 29) = 18.25, *p* = 0.0002, $\eta_{G}^{2}$ = 0.05. We again observed a significant interaction between pre-cue types, *F*(1, 29) = 6.00, *p* = 0.02, $\eta_{G}^{2}$ = 0.02. The SBA effect was only significant on feature-valid trials, *t*(29) = 5.41, *p* < 0.001, *d* = 0.99, and was insignificant on feature-invalid trials, *t*(29) = 1.01, *p* = 0.43, *d* = 0.18. The analogous pattern existed for the FBA effect as well: space-valid, *t*(29) = 4.99, *p* < 0.001, *d* = 0.91; space-invalid, *t*(29) = 0.30, *p* = 0.98, *d* = 0.0055. Thus, non-decision time was reduced only when all aspects of the pre-cue were valid.

In the current experiment, perceptual decision making primarily benefited from valid spatial cues. Given the nonequivalent attention effects in accuracy and RT across space and feature cues, we conclude that participants heavily relied on spatial cues to enhance the target signal during the task, even though both cues were equivalently reliable and independently effective (see Experiment 1). This finding was further supported by gap size estimates and the drift rate outputs of the robust EZ-diffusion model. FBA was engaged, however, in processes outside of signal enhancement. When combined with a valid spatial cue, valid feature cues facilitated the speed with which evidence accumulation onset and in turn elicited relatively conservative responses, without improving the resolution in the directed search area.

### Comparisons between experiments

Thus far, we have argued that, in the current experiment, SBA was used primarily to enhance the perceptual representation of the target, and FBA influences were largely restricted to decision making processes outside of signal enhancement. This conclusion is supported in part by the accuracy results presented, in which we did not observe an FBA effect, nor an interaction between cue types. The latter result is consistent with Experiment 2 of White et al. (2015), in which the authors argue that the absence of an interaction between cue types is indicative of independent space and feature selection mechanisms. However, the surprisingly absent FBA effect in accuracy observed here, when considered alone, makes it difficult to draw conclusions about a possible interactive relationship between mechanisms. Because we observed FBA effects in accuracy when the feature cue was presented alone in Experiment 1, it is possible that our data reflect a form of interdependence among selection mechanisms, albeit one where the deployment of one precludes the deployment of the other for the purposes of target identification.

Given that the patterns between Experiments 1 and 2 largely differed, we next conducted statistical comparisons to determine if these differences were meaningful. These comparisons serve two goals. First, we may be able to draw firmer conclusions about possible signal enhancement-specific dependencies between selection mechanisms when contrasting single cue and dual cue conditions. Thus, we focused primarily on comparing the lone valid cue conditions from Experiment 1 to both the single valid and wholly valid cue conditions from Experiment 2. Note that this analysis allows us to secondarily investigate any potential benefits and/or costs associated with combining two cues. All *p* values reported here were subjected to FDR correction to ward against an inflated type I error resulting from multiple comparisons.

#### Feature cue

There was a signal enhancement cost associated with pairing a valid feature cue with an invalid spatial cue as reflected in *d′*, *t*(42) = 3.35, *p* = 0.0069, *d* = 1.06, and drift rate, *t*(42) = 3.45, *p* = 0.0051, *d* = 1.09. A cost also existed in RT, *t*(42) = 3.03, *p* = 0.006, *d* = 0.98. At the same time, pairing a valid feature cue with a valid spatial cue did not result in a detectable benefit in any of these measures: *d′*, *t*(42) = 1.13, *p* = 0.53, *d* = 0.37; drift rate, *t*(42) = 1.64, *p* = 0.22, *d* = 0.53; RT, *t*(42) = 1.42, *p* = 0.16, *d* = 0.45. In fact, when we compared FBA effects (feature valid – feature invalid), we found they were significantly larger in both accuracy and drift rate when the feature cue was presented alone than when it was paired with a valid spatial cue: (*d′*, *t*(42) = 4.78, *p* < 0.0001, *d* = 1.55; drift rate, *t*(42) = 4.35, *p* < 0.001, *d* = 1.36. Note that this pattern runs counter to independent model predictions of additive effects manifested as statistically equivalent FBA effects regardless of the presence or validity of a spatial cue. Instead, our result suggests that SBA may have been used for signal enhancement at the exclusion of FBA in Experiment 2.

There was no change in boundary separation when a valid feature cue was paired with an invalid spatial cue, *t*(42) = 0.19, *p* = 0.85, *d =* 0.06, but responses were significantly more conservative with the addition of a valid spatial cue, *t*(42) = 4.18, *p* < 0.001, *d =* 1.29 (albeit with no concurrent change in accuracy). The addition of either an invalid or valid spatial cue had no detectable impact on non-decision time: invalid spatial cue, *t*(42) = 1.74, *p* = 0.12, *d =* 0.61; valid spatial cue, *t*(42) = −0.58, *p* = 0.57, *d =* –0.20.

#### Spatial cue

The analogous analyses described, applied to the spatial cue, produced almost entirely opposite patterns. In this case, there was no cost in signal enhancement when a valid spatial cue was paired with an invalid feature cue: *d′*, *t*(42) = 0.58, *p* = 0.56, *d =* 0.20; drift rate, *t*(42) = 0.46, *p* = 0.65, *d =* 0.16. Similarly, the addition of a valid feature cue did not elicit an increase in signal enhancement compared with a lone valid spatial cue: *d′*, *t*(42) = 0.85, *p* = 0.54, *d* = 0.28; drift rate, *t*(42) = 0.85, *p* = 0.53, *d =* 0.27. In fact, SBA effects in both measures were statistically matched when the spatial cue was presented alone or paired with a valid feature cue: *d′*, *t*(42) = 1.59, *p* = 0.12, *d* = 0.52; drift rate, *t*(42) = 1.24, *p* = 0.22, *d* = 0.41. Thus, unlike the pattern observed for FBA, the size of the SBA effect within accuracy and drift rate did not depend on the presence or validity of a concurrent feature cue.

There was, however, a cost in RT, *t*(42) = 3.90, *p* = 0.0014, *d =* 1.35, and non-decision time, *t*(42) = 4.26, *p* < 0.001, *d =* 1.53, when a valid spatial cue was paired with an invalid feature cue, as well as a decrease in boundary separation, *t*(42) = 2.44, *p* = 0.025, *d =* 0.81. An invalid feature cue led to slower non-decision time and, in turn, less conservative responses. Somewhat surprisingly, we also observed a relative cost in RT when a valid spatial cue was paired with a valid feature cue, *t*(42) = 3.01, p = 0.006, *d =* 1.04, and a marginal cost in non-decision time, *t*(42) = 2.27, p = 0.056, *d =* 0.81, albeit of smaller magnitudes than what was observed with a paired invalid feature cue (see Experiment 2 Results). Note that a similar relationship did not exist when we considered feature cues above, indicating that the inclusion of two relevant cues instead of one did not broadly increase non-decision time. Instead, this dimension-specific cost may be accounted for by the relatively longer non-decision time associated with the feature cue (see Experiment 1 Results and Discussion).

The sum of these results indicate that SBA was largely used to resolve the target, whereas FBA was relegated primarily to perceptual decision processes outside of signal enhancement.

## General discussion

The present study investigated possible interactions between SBA and FBA by providing both spatial and feature information via an endogenous pre-cue to facilitate upcoming target identification in a sparse display. Notably, in this task both a valid location and color cue would redundantly filter out the same distracting items while directing attention to the same relevant stimulus. Thus, we can explore whether SBA and FBA combinatorially improve the perceptual decision making of a single item.

Attention effects within accuracy are typically larger in cluttered displays in which nearby distractors compete for selection with the target, where signal enhancement and distractor suppression operate in tandem to facilitate perceptual processing of a target (Awh, Sgarlata, & Kliestik, 2005; Carrasco, 2011), and can be difficult to detect in sparse displays (Dosher & Lu, 2000; Grindley & Townsend, 1968; Scolari & Awh, 2019; Shiu & Pashler, 1994). As such, attention effects within sparse displays have typically been restricted to RT (Egner et al., 2008; Kingstone, 1992; Lambert & Hockey, 1986; but see Bengson et al., 2012), precluding a strong test of signal enhancement. Here, we ensured the uncluttered relevant item was nonetheless nonsalient by titrating the size of the target gap to keep accuracy within a prescribed range, thus, revealing attention effects in both behavioral measures. In addition to analyzing RT and accuracy separately, keeping performance below ceiling allowed us to use a simple diffusion model to further investigate selective attention influences on unique components of perceptual decision making.

### Signal enhancement effects

In Experiment 1, we demonstrated that our sparse display design can elicit detectable selective attention effects of similar magnitudes following either a location or color pre-cue in both accuracy and RT. Furthermore, the titration procedure we used to match task difficulty across participants produced similar gap sizes across cue types, indicating equal effectiveness. These patterns were corroborated by the drift rate estimates produced by the diffusion model: for both cue types, the speed of evidence accumulation on correct trials was significantly faster after valid compared with invalid pre-cues. Based on these results, we conclude that each endogenous cue was used effectively to resolve the target, when presented in isolation.

Given that SBA and FBA independently showed a similar degree of perceptual facilitation in Experiment 1, we next set out to characterize possible interactions between both selection mechanisms using an integrated space and feature cue with the identical stimulus display. Surprisingly, our findings indicate that only the spatial cue, and not the feature cue, was used to perceptually resolve the target gap. This finding is indicated in our analysis of performance accuracy: only an SBA effect was significant, and the size of the effect did not increase when paired with a concurrent valid feature cue. Similarly, when compared with Experiment 1 in which one of the two relevant dimensions was never cued for each participant, pairing a valid spatial cue with an invalid feature cue did not detrimentally affect accuracy. The same pattern held across cueing conditions within estimated drift rates. Together, these results suggest that, across all participants, FBA did not impact the rate at which evidence was accumulated in favor of a correct response, nor did it impact response selection.

White et al. (2015) investigated how top-down SBA and FBA jointly influence perception in a task using superimposed dot patterns. They concluded that, when deployed together, SBA and FBA additively enhance visual signals, consistent with an independent systems model. In contrast, while using a sparse display and a perceptually demanding task, we were unable to find evidence of additive (or super-additive) enhancement, despite our expectations. This difference may be due in part to the absent FBA effect reported in Experiment 2. Note that models of selective attention that assume either independent or dependent mechanisms would predict the same pattern of results observed here if one mechanism was not deployed. However, a strictly independent model would also predict that if FBA is effective alone, as was demonstrated in Experiment 1, it should be used in signal enhancement regardless of the presence or validity of a concurrent spatial cue. Here, our results depart from this expectation: When we compared FBA effects across experiments, we found that it was significantly smaller in Experiment 2 (note again that this was not the case for the SBA effect). Thus, FBA effects were convincingly absent only when a feature cue was paired with a spatial cue. This finding suggests some interdependence between mechanisms, whereby the deployment of spatial selection to enhance the target signal may have precluded similar deployment of feature selection.

### Higher order effects

Although our results do not conform to the expectations of strictly independent models, it is important to note that neither are they necessarily at odds with White et al. (2015). Top-down visual attention is broadly subdivided into two interactive components: attentional control, localized to a broad swath of frontoparietal cortex, and attentional modulation of ongoing visual processing within sensory cortex (Scolari, Seidl-Rathkopf & Kastner, 2015). Behavioral evidence of signal enhancement likely is the result of the latter. It is possible, then, that space-based and feature-based modulatory signals occurring within visual cortex are independent in nature (Hayden & Gallant, 2009; McAdams & Maunsell, 2000), whereas a domain-general control center governs such modulations (Andersen et al., 2011; Scolari et al., 2014; Scolari et al., 2015). Consistent with this possibility, several studies have demonstrated that a common network of frontoparietal regions are activated during space-, feature-, and object-based attention tasks (Corbetta & Shulman, 2002; Greenberg et al., 2010; Ibos & Freedman, 2016; Liu, Hospadaruk, Zhu, & Gardner, 2011; Serences, Schwarzbach, Courtney, Golay, & Yantis, 2004; Shomstein & Behrmann, 2006). In that case, in our experiment, control centers may have generated modulatory signals on the basis of space alone, while restricting such signals in response to the feature cue. This notion leaves open the possibility of independent, additive effects within signal enhancement when a domain-general attentional control system generates modulatory signals in accordance with both selection mechanisms.

The possibility of a higher order dependency between space- and feature-based selection mechanisms is supported when we consider components of perceptual decision making outside of signal enhancement. First, when considering RT as a unitary measure, we found a significant FBA effect only when the spatial cue was valid (in line with Bengson et al., 2012; Soto & Blanco, 2004; Stoppel et al., 2007), and similarly, the spatial cueing effect was numerically larger when paired with a valid feature cue. However, although other studies have reported significant interactions within RT between cue type and cue validity in similarly sparse displays (Bengson et al., 2012; Kingstone, 1992), the patterns observed here did not reach significance. On the face of it, our RT results may be more in line with Lambert and Hockey (1986), where over the course of four experiments and seven testing sessions, apparent trends toward interactions between SBA and FBA only reach significance in one scenario; as well as Egner et al. (2008), who reported only additive effects within RT.

Notably, however, RT is likely indicative of a multitude of perceptual decision-making processes and, as such, may obscure differential patterns within signal strength, response conservativeness and processing speed (Ratcliff, 1978; Wagenmakers et al., 2007; White et al., 2015), thus potentially accounting for some of the disparate patterns in the literature. When we subjected our response time and accuracy data to a robust EZ-diffusion model to separately estimate each of three unobservable component parts (Wagenmakers et al., 2008), we found compelling evidence for an interactive relationship between SBA and FBA within perceptual decision-making processes outside of signal strength. The amount of time spent before evidence accumulation (non-decision time) was shortest when both dimensions of the cue were valid, and both spatial cueing and feature cueing effects only emerged when the other dimension was valid. Response conservativeness (or boundary separation) mirrored these results, suggesting that reduced non-decision time allowed for more time spent engaged in evidence accumulation without a concurrent change in accumulation rate. Thus, super-additive effects, reflecting dependency between mechanisms, were present within perceptual decision making (Andersen et al., 2011). Importantly, these findings may shed light on why many behavioral studies that support a model of dependency between selection mechanisms more frequently (albeit not exclusively) rely on RT evidence, whereas those that claim independency more frequently rely on accuracy (e.g., Bengson et al., 2012; Kingstone, 1992; White, Rolfs, & Carrasco, 2013; 2015).

Interestingly, although a wholly valid cue exhibited a reduced non-decision time compared with a valid–invalid cue, pairing a valid spatial cue with a feature cue, regardless of its validity, resulted in a significantly longer non-decision time than a spatial cue alone (see Comparisons between Experiments). The estimate for the dual cue was matched with the non-decision time estimated for a lone feature cue. This finding is consistent with previous demonstrations that have shown engaging FBA may be sluggish relative to SBA. Importantly, the cue-to-target SOAs used in the current study exceeded the expected duration needed to effectively deploy FBA (Liu et al., 2007; see Experiment 1). Therefore, the relative speed reduction observed here is unlikely attributable to the interpretation of, and attentional preallocation in response to, the pre-cue. Rather, it may be reflective of the finding that FBA effects emerge on a relatively slower timescale after stimulus display onset, even without the need to interpret and respond to a trial-by-trial feature cue (Anllo-Vento & Hillyard, 1996). As we speculate in the Results and Discussion of Experiment 1, this finding may be a consequence of a global deployment of FBA across space (Liu et al., 2007; Rossi & Paradiso, 1995; Serences & Boynton, 2007).

Importantly, the results of the diffusion model demonstrate that participants were not simply ignoring the feature cue in Experiment 2. Instead, the feature cue was likely used to help determine whether the correct item was within the focus of attention before evidence accumulation; once participants began accumulating evidence for one of the two response alternatives, though, FBA influences were uniformly and convincingly absent. In contrast, evidence of SBA was present in all aspects of perceptual decision making.

### Single versus dual deployment of selection mechanisms

Although our data are well accounted for via a descriptive model of higher order dependency between mechanisms, we are left to puzzle about why FBA effects did not emerge, despite expectations, within indirect measures of signal enhancement in Experiment 2. First, we consider in general why only one cue might be used for this purpose when two are available. We then turn to the specific absence of an FBA effect in favor of SBA.

In the current task design, we set the pre-cue to be wholly valid on 70% of all trials under the assumption that if both selection mechanisms could be deployed simultaneously to (super-)additively improve the perceptual resolution of the target gap, participants should be motivated to do so given the impact it would have on a large majority of trials. However, we failed to detect dual deployment across all participants. Even among those who exhibited both SBA and FBA effects in accuracy, target gap size was not reliably different from those who only exhibited SBA effects. This pattern of results indicates that either the former subset of participants switched between selection mechanisms from trial to trial and thus failed to deploy both simultaneously, or that deploying both mechanisms simultaneously did not improve perceptual resolution.

Following the findings and logic of White et al. (2015), we suspect that the low stimulus competition used in the present study could in part account for this result. In their design, a relevant dot pattern was defined both by its color and location, each of which was shared with irrelevant dot patterns. Thus, the feature and space components of the endogenous cue served to both 1) direct attention to a particular color and location (signal enhancement), and 2) filter out irrelevant colors at the attended location and irrelevant locations containing the relevant color (distractor suppression). Thus, deploying both selection mechanisms in response to a wholly valid cue decreased the amount of attended information. Furthermore, attention effects were super-additive only when the irrelevant dot patterns were highly salient, presumably operating simultaneously to decrease stimulus competition. In our experiment, no distractors shared color or location information with the relevant item, and both components of the frequent, wholly valid cue directed attention to the same, single item (signal enhancement without distractor suppression).

Given that we did not see additive effects between SBA and FBA, but rather an apparent nearly complete reliance on just the spatial cue to enhance the target signal, we suggest that such dual independent deployments may only emerge when the two selection mechanisms make sufficiently unique contributions to identifying the target. If top-down selection is limited to (largely redundant) signal enhancement, it may be the case that only one mechanism is deployed for this purpose even when cues for two (or more) are available. The high frequency of the wholly valid cue, where either cue type is equally predictive, and the limited benefit of using both dimensions to resolve the target, may have prompted participants to strategically use only one throughout the task. This plausible account is consistent with our suggestion that higher order control centers may have generated space-based modulatory signals in visual cortex while restricting analogous feature-based signals.

We argue that participants deployed one attention mechanism nearly unilaterally to resolve the perceptually demanding target in the sparse display, and that deploying both to a single item may be of limited benefit to enhancing the signal. We next consider why participants largely preferred the spatial cue, specifically, over the feature cue, despite our expectations that both should be equally viable. Although we used a localization task, neither cued dimension predicted the to-be-reported target gap. The arrow cue was never fully aligned with the two response alternatives (right vs. left), given that it directed attention to the center of one of four quadrants (upper right, lower right, upper left, or lower left). Furthermore, on approximately one-half the trials, the horizontal aspect (left or right) of the arrow cue conflicted with the object-based location of the target gap. Nonetheless, the Experiment 2 results raise the possibility that the spatial cue was more closely related to our localization task. This finding is consistent with the speculation regarding non-decision time offered in Experiment 1 Results and Discussion: following pre-allocated FBA, only the subset of modulated sensory neurons whose receptive fields included the relevant item would be informative for locating the gap. In contrast, all sensory neurons modulated by SBA, regardless of color selectivity, should carry at least some information for the task. Thus, although the participants used FBA effectively in Experiment 1, they may have strategically used only the more useful SBA when both cue types were available.

A potential caveat to this explanation is that Experiment 1 demonstrated that both cue types were equally effective in resolving the target—as reflected in accuracy, gap size, and drift rate—when presented alone. This finding suggests that the advantage of space-based selection exists just before evidence accumulation. Yet, participants exhibited a prolonged non-decision time in Experiment 2, presumably to use feature information to verify that the relevant item was attended. A second possibility is that faster onset time of SBA (Anllo-Vento & Hillyard, 1996) led participants to detect its effectiveness at resolving the target more easily than the feature cue, despite both being equally effective. Similarly, onset speed of evidence accumulation may have impacted implicit statistical learning of regularities in the task (Zhao, Al-Aidroos, & Turk-Browne, 2013), leading to differences in the probabilistic expectancy between the two types of cues (Mordkoff & Yantis, 1991). This finding would suggest that cue preference should be modulated by cue validity (a factor that we did not manipulate here). Whether this post hoc speculation holds is an intriguing question for future research.

## Conclusion

Our results show that both FBA and SBA can be used in isolation to resolve a perceptually challenging target in a sparse display. However, there may be little signal enhancement benefit to voluntarily deploying both simultaneously in the absence of stimulus competition. Instead, we showed that both mechanisms conjunctively facilitate components of decision making, while only spatial selection enhanced the target representation. This suggests at least some higher order interdependence between mechanisms.

## Supplementary Material

Supplement 1

Supplement 2
