# Supplementary material for: Limited interactions between space- and feature-based attention in visually sparse displays
Source: J Vis. 2020 Apr 9;20(4):5. doi: 10.1167/jov.20.4.5 (PMC7405816; doi:10.1167/jov.20.4.5)
Supplement: Supplement 1 [file jovi-20-4-5_s001.docx]

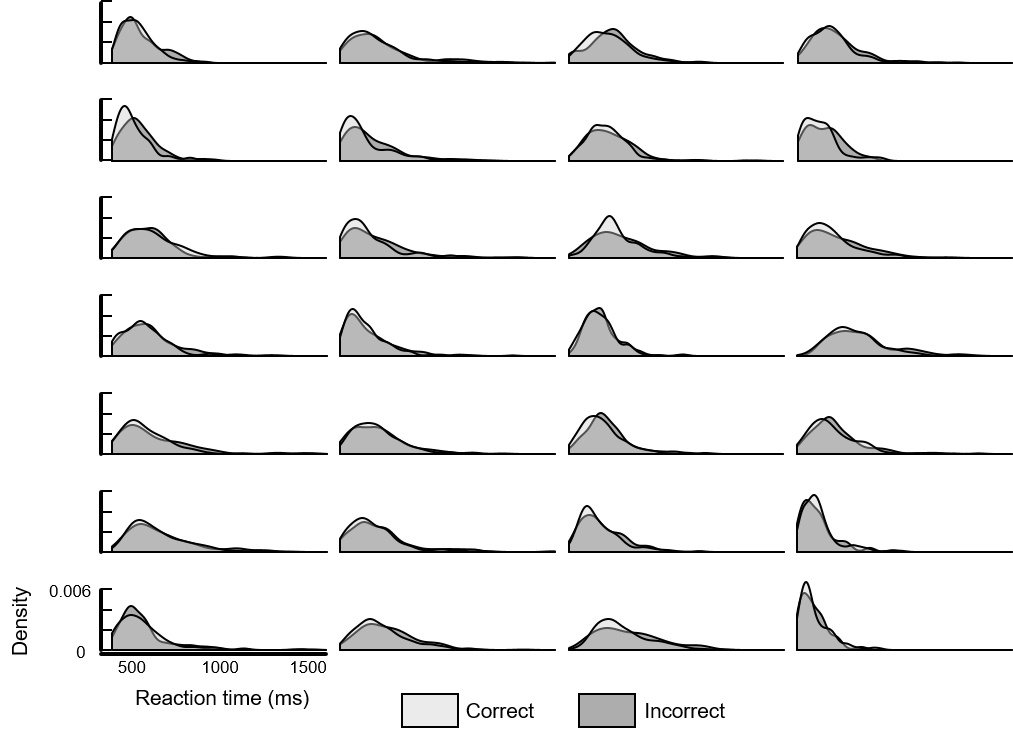


Supplementary Figure S1. Reaction time distributions for correct and incorrect responses from Experiment 1. Each plot represents data from a single participant.


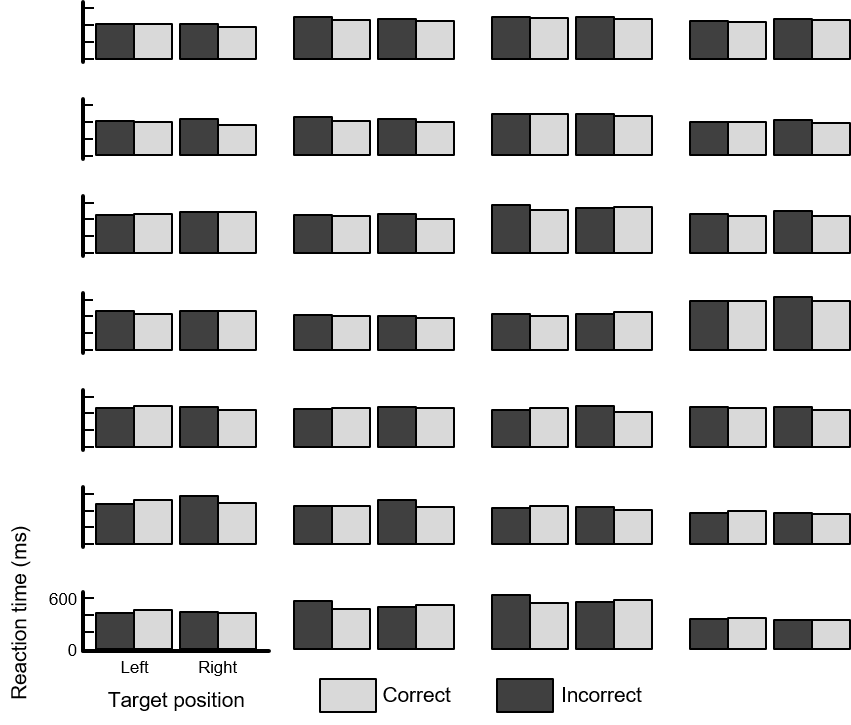


Supplementary Figure S2. Mean reaction times for correct and incorrect responses for each stimulus alternative (target gap positioned on the left or right) from Experiment 1. Each plot represents data from a single participant.


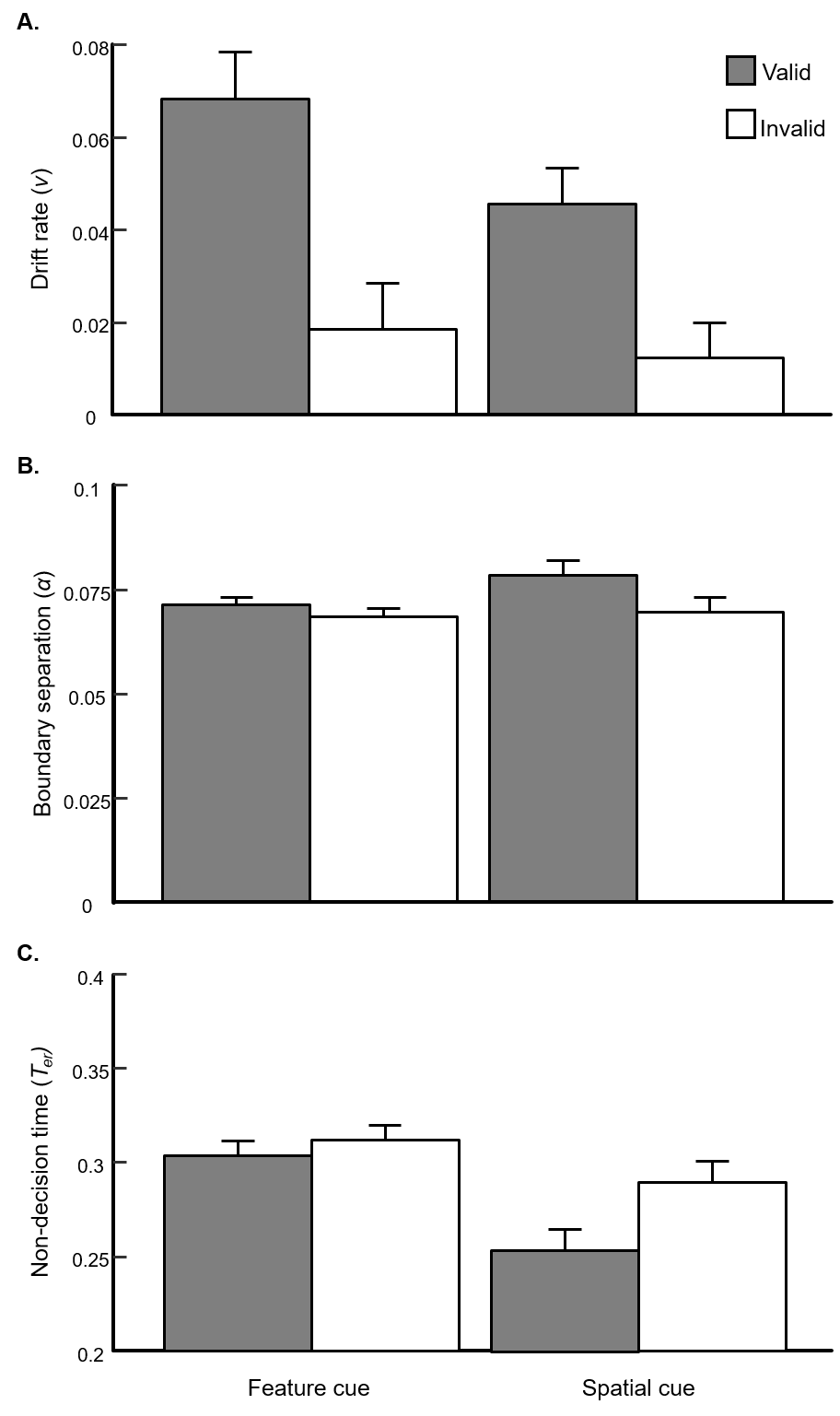


Supplementary Figure S3. Results from the robust EZ-diffusion model are plotted for both pre-cue groups from Experiment 1; 4 participants were removed for violating at least one assumption of the model. Drift rate (A), boundary separation (B) and non-decision time (C) are plotted for valid and invalid trials separately for each group. Error bars reflect ±1 within-participant SEM.


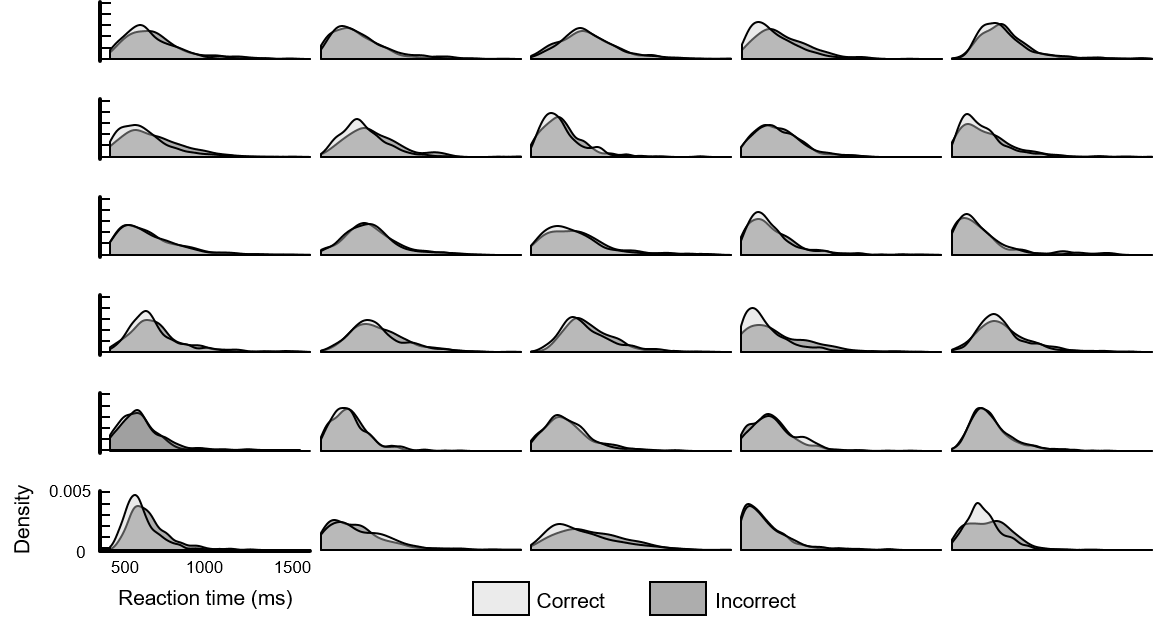


Supplementary Figure S4. Reaction time distributions for correct and incorrect responses from Experiment 2. Each plot represents data from a single participant.


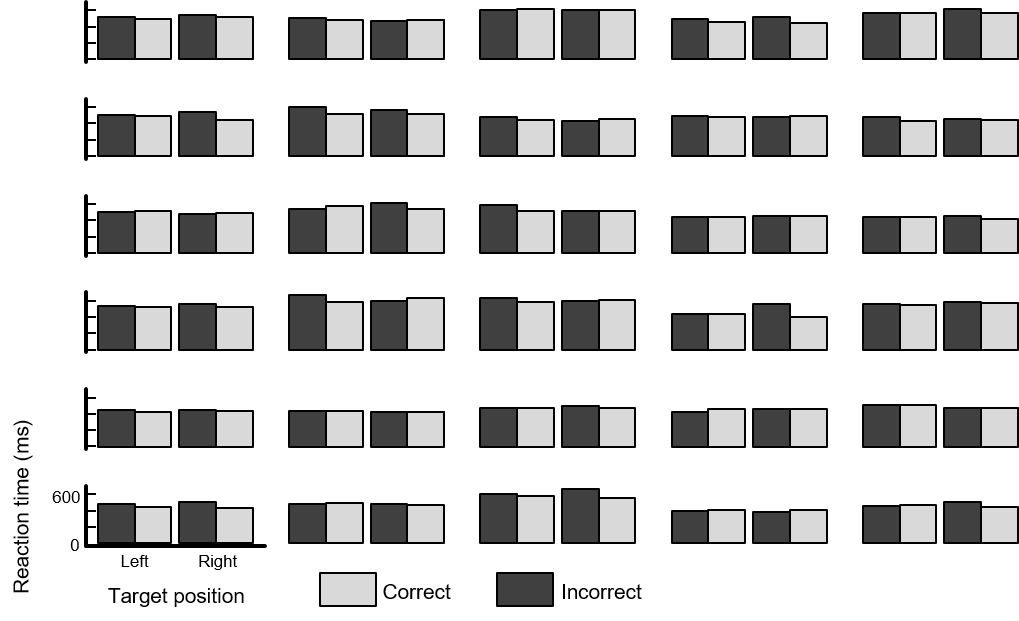


Supplementary Figure S5. Mean reaction times for correct and incorrect responses for each stimulus alternative (target gap positioned on the left or right) from Experiment 2. Each plot represents data from a single participant.


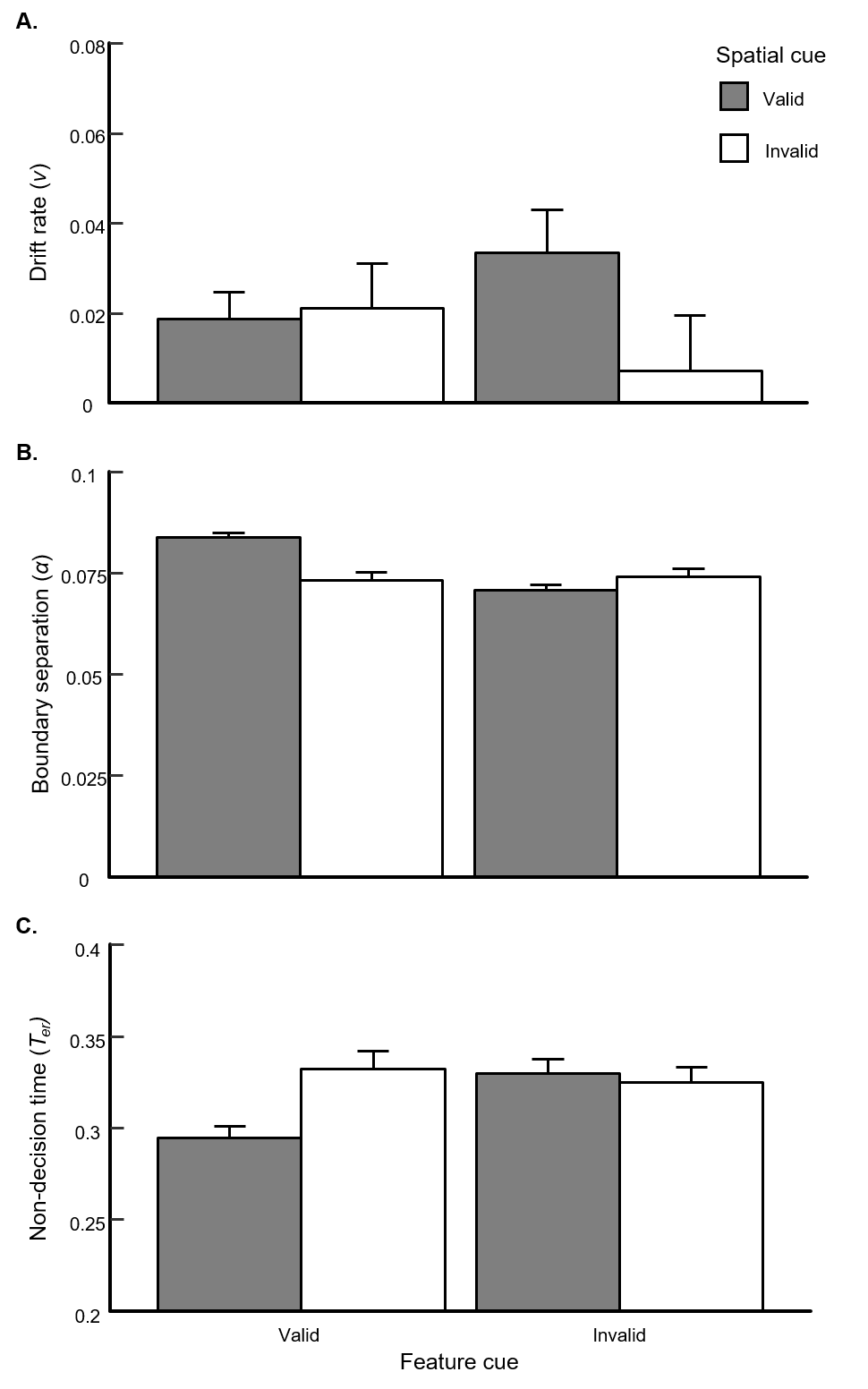


Supplementary Figure S6. Results from the robust EZ-diffusion model are plotted for Experiment 2; 12 participants were removed for violating at least one assumption of the model. Drift rate (A), boundary separation (B) and non-decision time (C) are plotted for valid and invalid trials separately for each cue type. Error bars reflect ±1 within-participant SEM.
