# Supplementary material for: Limited interactions between space- and feature-based attention in visually sparse displays
Source: J Vis. 2020 Apr 9;20(4):5. doi: 10.1167/jov.20.4.5 (PMC7405816; doi:10.1167/jov.20.4.5)
Supplement: Supplement 2 [file jovi-20-4-5_s002.docx]

Supplementary Material for Experiment 1

Four participants from Experiment 1 violated at least one assumption of the robust EZ-diffusion model (see *Results & Discussion* in the main text for more details). Here, we report the output of the model (drift rate, boundary separation, and non-decision time) with those individual participants removed to verify its robustness against few violations. All conclusions remain unchanged whether the four participants are included.

*Drift rate.* When collapsing across cue type, drift rate was significantly greater following a valid pre-cue (M = 0.06) than an invalid pre-cue (M = 0.02), *F*(1, 22) = 21.77, *p* < 0.001, $\eta_{G}^{2}$ = 0.22. This pattern was significant for both the FC group, *t*(11) = 3.52, *p* = 0.005, *d* = 1.02, and the SC group, *t*(11) = 3.08, *p* = 0.010, *d* = 0.90. Consistent with the results reported in the main text, we did not observe a main effect of cue type, *F*(1,22) = 1.07, *p* = 0.31, $\eta_{G}^{2}$ = 0.03, nor an interaction between cue type and cue validity, *F*(1,22) = 0.86, *p* = 0.36, $\eta_{G}^{2}$ = 0.01. The rates of evidence accumulation were thus well matched for both valid spatial and valid feature cues (see Supplementary Figure S3A).

*Boundary separation.* We observed a marginal main effect of cue validity in boundary separation, *F*(1,22) = 4.01, *p* = 0.058, $\eta_{G}^{2}$ = 0.07, with more conservative responses occurring on valid trials (M = 0.075) than invalid trials (M = 0.069). Just as we report in the main text, this pattern was not observed when considering either pre-cue group alone (FC group: *t*(11) = 1.03, *p*=0.326, *d* = 0.30; SC group: *t*(11) = 1.72, *p* = 0.114, *d* = 0.50). We did not observe a main effect of cue type, *F*(1,22) = 1.63, *p* = 0.214, $\eta_{G}^{2}$ = 0.03, nor an interaction between cue type and cue validity, *F*(1,22) = 0.86, *p* = 0.318 (see Supplementary Figure S3B). This pattern is fully consistent with the results reported in the main text. Thus, we maintain our conclusion that responses were relatively more conservative on valid than invalid trials.

*Non-decision time.* Participants on average spent more time preparing for evidence accumulation following an invalid cue (M = 0.30) than a valid cue (M = 0.28), *F*(1,22) = 5.30, *p* = 0.031, $\eta_{G}^{2}$ = 0.07 (see Supplementary Figure S3C). This pattern held when considering the SC group alone, *t*(11) = -2.28, *p* = 0.044, *d* = -0.66, but not for the FC group, *t*(11) = -0.75, *p* = 0.466, *d* = -0.22. Non-decision time for the SC group (M = 0.27) was also generally shorter than the FC group (M = 0.31), *F*(1,22) = 5.96, *p* = 0.023, $\eta_{G}^{2}$ = 0.16. However, there was no significant interaction between cue type and cue validity, *F*(1,22) = 2.09, *p* = 0.162, $\eta_{G}^{2}$ = 0.03. This pattern of results is again fully consistent with what we observed when considering the full dataset.

Supplementary Material for Experiment 2

Twelve participants from Experiment 2 violated at least one assumption of the robust EZ-diffusion model (see *Results & Discussion* in the main text for more details). Here, we report the output of the model (drift rate, boundary separation, and non-decision time) with those individual participants removed to verify the robustness of the model. Reducing our dataset from 30 participants to 18 consequently also reduced our power to detect significant effects. Importantly, however, the effects reported in the main text still held for most model components (with the exception of drift rate and a main effect of the feature cue in non-decision time; see below), and no previously unreported effects emerged. Where the results failed to reach significance, the trends nonetheless largely converged with the full dataset. Thus, the conclusions drawn in the main text still stand.

*Drift rate.* Using only the 18 participants who did not exhibit any violations to the model assumptions, drift rate did not reliably differ between valid and invalid trials for either the feature cue, *F*(1,17) < 0.01, *p* = 0.968, $\eta_{G}^{2}$ < 0.001, nor the spatial cue, *F*(1,17) = 1.43, *p* = 0.248, $\eta_{G}^{2}$=0.02). Note that the absence of an SBA effect is a departure from the results reported in the main text. Although the drift rate data depicted in Supplementary Figure S6A suggest a possible cross-over interaction between cue types, it did not reach significance, *F*(1,17) = 2.12, *p* = 0.164, $\eta_{G}^{2}$ =0.03. Similarly, there was no detectable interaction when considering the full dataset. We are nonetheless inclined to consider the possibility that the failure to detect an interaction here could be the result of low power, given the depicted trend and small effect size (Bakeman, 2005). Thus, we considered the SBA effect for feature-valid and feature-invalid trials separately, and we did not observe a reliable cueing effect in either case, (feature-valid: *t*(17) = -0.21, *p* = 0.838, *d* = 0.05; feature-invalid: *t*(17) = 1.58, *p* = 0.132, *d* = 0.37). More importantly, though, the trend shows that the mean SBA effect was numerically *smaller* when the feature cue was valid than when it was invalid, which is fully inconsistent with a super-additive dependency between SBA and FBA. Therefore, whether the interaction is absent or underpowered, the results do not conform to traditional dependent models.

*Boundary separation.* With this reduced dataset, participants on average made more conservative responses following a valid feature pre-cue (M = 0.078) than following an invalid feature pre-cue (M = 0.073), resulting in a significant FBA effect, *F*(1,17) = 11.41, *p* = 0.004, $\eta_{G}^{2}$ = 0.09. An analogous SBA effect was also observed (valid spatial pre-cue: *M* = 0.077; invalid spatial pre-cue: *M* = 0.074), *F*(1,17) = 9.96, *p* = 0.006, $\eta_{G}^{2}$ = 0.03. Critically, there was a significant interaction between cue types, *F*(1,17) = 12.45, *p* = 0.003, $\eta_{G}^{2}$ = 0.11, echoing the results produced when considering the full dataset (see Supplementary Figure S6B).

*Non-decision time.* Although we observed a main effect of the feature cue in non-decision time with all 30 participants included in the analyses, the results fell short of significance here, *F*(1,17) = 3.03, *p* = 0.100, $\eta_{G}^{2}$ = 0.01. However, the same trend was present, with non-decision time being numerically shorter following a valid feature cue (*M* = 0.313) compared to an invalid feature cue (*M* = 0.327). The main effect of the spatial cue still held, with shorter non-decision time following a valid spatial cue (*M* = 0.312) compared to an invalid spatial cue (*M* = 0.328), *F*(1,17) = 4.47, *p* = 0.050, $\eta_{G}^{2}$ =0.02. Importantly, we observed an interaction between spatial and feature cues, *F*(1,17) = 6.25, *p* = 0.023, $\eta_{G}^{2}$ = 0.03, consistent with the results reported in the main text (see Supplementary Figure S6C). Non-decision time was shortest when both pre-cues were valid. The pattern observed for non-decision time largely mirrors that observed for boundary separation, as discussed in the main text.

References

Bakeman R. (2005). Recommended effect size statistics for repeated measures designs. *Behavior Research Methods*, 37, 379–384. doi: https://doi.org/10.3758/BF03192707
